# The impact of Medical Associate Professions (MAPs) on the productivity, quality of care, patient and healthcare workforce satisfaction, and budget implications in various healthcare settings: a systematic review

**DOI:** 10.1186/s12913-025-13626-4

**Published:** 2025-11-19

**Authors:** Dmytro Babelyuk, Vladyslav Kulikov, Llinos Haf Spencer, Deborah Fitzsimmons, Rhiannon Tudor Edwards

**Affiliations:** 1 Centre for Health Economics and Medicines Evaluation (CHEME), Bangor University, Holyhead Road, Ardudwy, Bangor, Gwynedd, LL57 2PZ Wales, UK; 2 Welsh Institute for Health and Social Care (WIHSC), Faculty of Life Sciences and Education, University of South Wales, Pontypridd, CF37 4BD Wales, UK; 3 Faculty of Nursing and Midwifery, Royal College of Surgeons in Ireland, University of Medicine and Health Sciences, 123 St Stephen’s Green, Dublin, D02 YN77 Ireland; 4 Swansea Centre for Health Economics (SCHE), Swansea University, Swansea, SA1 8EN Wales, UK

**Keywords:** Physician Associate (PA), Anaesthetic Associate (AA), Surgical Care Practitioner (SCP), Quality of care, Cost-effectiveness, Patient satisfaction, Healthcare workforce perceptions, Productivity

## Abstract

**Background:**

Healthcare staff planning in the NHS in the UK has undergone significant changes in recent years, driven by declining productivity, staff shortages, and rising patient demand. Innovative staff planning decisions include implementing new non-medical roles, collectively referred to as “Medical Associate Professions” (MAPs). The MAP roles were established in 2014 and encompass Physician Associates (PAs), Anaesthetic Associates (AAs), and Surgical Care Practitioners (SCPs). This systematic review has been conducted to evaluate and synthesise international evidence on the impact of MAPs on health system productivity, quality of care, patient satisfaction, perceptions of the healthcare workforce regarding roles, and the budget implications of their implementation in various healthcare settings.

**Method:**

Electronic database searches were conducted using the Medline, Embase, CINAHL, Web of Science, and PubMed databases for studies published between 2004 and 2024. Blinded parallel processes were used to screen abstracts and full text of the studies that met the inclusion criteria. Data extraction, narrative synthesis and quality assessments were undertaken for the included studies. The impact on productivity, quality of care, patient and colleague perceptions, and cost-effectiveness of the roles were chosen as the key outcomes of interest.

**Results:**

A total of 8725 papers were identified following the systematic searching of the databases, and 69 papers were included in the review. These included cost-effectiveness analysis (*n* = 1), cross-sectional studies (*n* = 18), cohort studies (*n* = 29), qualitative studies (*n* = 9), case series studies (*n* = 1), case-control study (*n* = 1), and mixed-method studies (*n* = 10).

**Conclusions:**

MAPs have the potential to complement traditional workforce configuration to support productivity and quality of care. There is evidence that they are acceptable to patients, and there is mixed evidence about their contribution in the eyes of clinical colleagues. A modest amount of data is available on PAs and much less on SCPs and AAs. Despite most of the papers being of moderate to poor quality, our rigorous and innovative systematic review reflects the evidence that incorporating PAs and SCPs into healthcare can have a positive impact on productivity and the quality of care provided, reduce overall personnel and care costs, and elicit positive feedback from both patients and colleagues when appropriately implemented.

**PROSPERO registration number:**

CRD42023457692.

**Supplementary Information:**

The online version contains supplementary material available at 10.1186/s12913-025-13626-4.

## Introduction

The global shortage of healthcare workers has been a significant concern in recent years, and according to recent World Health Organisation (WHO) reports, the situation is unlikely to improve [1]. For example, by 2030, the European Union is expected to face a shortfall of 4.1 million healthcare workers despite an increase in the overall workforce [2]. In the UK, factors such as Brexit, the rising cost of living, and increased healthcare demands have further strained the healthcare workforce, intensifying pressure on current staff and the system [3]. These challenges restrict the capacity of the healthcare system to address increasing needs, which adversely affects productivity, as recent reports suggest that National Health Service (NHS) productivity is 20% lower than before the COVID-19 pandemic [4].

As a result of these challenges, the NHS has witnessed the emergence of new professional roles and their increased use within multidisciplinary teams [5]. This is an ongoing effort to keep providing safe, accessible, and high-quality care for patients in the circumstances of the widespread healthcare workforce shortage by introducing new non-medical roles.

Three such non-medical roles were grouped under the heading of “Medical Associate Professions” (MAPs) by Health Education England (HEE) in 2014, with the intention to work “towards a common education and training programme to support a route to statutory regulation” [6]. While there are significant differences in their clinical scope of practice, they share similarities in their career framework, education, and training. The three roles are Physician Associate (PA), Anaesthesia Associate (AA) and Surgical Care Practitioner (SCP).

The development of MAPs began in the 1960s in the USA, with physician PAs emerging to address critical shortages in primary care [7]. This model soon spread globally, with countries like Ghana, India, the UK, and Germany adopting similar roles by the early 21st century [8]. AAs followed a similar trajectory, originating in the USA academic centres to combat anaesthesia workforce gaps, and reaching the UK by the 1990s through structured training and competency frameworks [9]. SCPs, as well, evolved from urgent wartime necessity into formalised roles, supporting surgical teams with advanced clinical capabilities [10]. The global expansion of PA, AA, and SCP roles reflected a shared response to persistent healthcare workforce shortages, with the UK among the key adopters.

The MAPs are a part of the healthcare workforce and are trained to work as a part of a multidisciplinary team to provide enhanced service delivery [11]. They are trained to be capable of practising in various specialities and aim to offer continuity of care, especially in acute settings and GP practices. MAPs are called to work within their sphere of competence and support doctors by taking on tasks that do not require their expertise, thus freeing up their time to focus on more complex patient care. However, they have significant limitations to their practice in the UK, for example, they do not have prescribing responsibilities and at present, cannot request CT scans or X-rays [12].

PAs, AAs, and SCPs have been widely integrated into healthcare systems across the UK and other countries over the last few decades (although they may be identified under varying titles), making their presence increasingly common across diverse clinical settings. PAs work in GP practices, emergency departments, and hospital wards, managing patients with acute or chronic conditions, performing clinical assessments, and supporting diagnosis and treatment planning under the doctor’s supervision [13]. However, AAs primarily support anaesthetic teams in surgical settings, performing tasks such as anaesthesia induction, maintenance, and patient assessment under supervision, and may also work in critical care or A&E [14]. SCPs assist consultant surgeons by conducting pre- and post-operative care, participating in procedures, and handling surgical tasks within their defined scope of practice [15]. These roles were designed to enhance service capacity and flexibility, particularly in the face of global workforce shortages, to address the international healthcare workforce strategy, bridging workforce gaps and supporting multidisciplinary care across settings [16].

The latest NHS England Long-Term Workforce plan confirmed the direction to expand the skills of healthcare workers across different teams by upskilling and introducing new roles while developing existing ones to align with patient needs [3]. A significant part of the plan is to increase the number of training places for MAPs, particularly PAs, to over 1,500 by 2031/32. This means that over 1,400 PAs will be trained per year, leading to a workforce of 10,000 PAs by 2036/37. For AAs, the number of training places should be increased to 280 by 2031/32. SCPs have their own development plan due to their unique training pathway.

Although MAPs have grown significantly in the UK and worldwide, there is still a lack of evidence on their impact on productivity and quality of care, which this systematic review aims to address. Additionally, there is limited information on how patients and colleagues view these roles and their cost-effectiveness, which will also be examined in this review.

Previous systematic reviews within this area of research, have primarily focused on PAs, as they are the most numerous role within the “umbrella” term of MAPs. Recent studies identified in this systematic review have also examined evidence specifically from primary or secondary care settings [17–19], assessed the contribution of these roles in emergency departments [20, 21], and explored their cost-effectiveness [22]. Another recent systematic review evaluated the public perception of PAs in the UK and acknowledged that, despite limited information about and understanding of the PA role, patients were largely satisfied with the quality of care they received from PAs [23].

MAPs, particularly PAs and AAs, have recently gained significant attention in social media, political discussions, and healthcare settings [24]. The complexities surrounding this controversy are explored in a recent paper by McKee et al. The authors pointed out several concerns about this emerging occupational group, which can be categorised into six main areas, including patient safety, scope of practice, informed consent, preferential employment conditions, additional workload, and effects on medical training [25]. Consequently, in late 2024, the Secretary of State for Health and Social Care, Wes Streeting, commissioned a review to assess the evidence regarding the safety and effectiveness of the roles which Gillian Leng is currently leading. Professor Trisha Greenhalgh published a recent rapid systematic review on PAs and AAs to address the issue and contribute to the ongoing nationwide study [26].

Despite that attention, the Department of Health and Social Care (DHSC) and General Medical Council (GMC) have recently introduced new legislation to regulate physician associates and anaesthesia associates [27]. This systematic review aims to fill the research gap concerning the impact of MAPs on healthcare delivery in the UK by providing evidence on their roles from an international perspective.

This systematic literature review assesses the current evidence on the contributions of all three roles within the MAPs healthcare staff group, including patient and colleague perceptions, as well as the budget implications of MAPs implementation in various international healthcare systems, as well as in the UK. It aims to fill the research gap concerning the impact of MAPs on healthcare delivery by providing updated international evidence. This evidence will contribute to informing both the UK-specific context policies and the global healthcare workforce market challenges.

This systematic review will address the following questions:

1. What is the impact of MAPs on the quality of care and productivity in primary, secondary and tertiary healthcare settings?
2. What is the perception of healthcare workforce colleagues towards MAPs?
3. What is patient satisfaction with received care and willingness to be seen by MAPs?
4. Are MAPs cost-effective, and what are the budget implications of implementing these roles to the multidisciplinary team in a primary, secondary or tertiary healthcare setting?

## Method

### Search strategy

This systematic review was conducted in accordance with international guidelines of Preferred Reporting Items for Systematic Reviews and Meta-Analyses (PRISMA) [28]. The complete search strategy is outlined in the research protocol registered with the International Prospective Register of Systematic Reviews (PROSPERO), with the CRD number CRD42023457692.

Systematic searches for relevant studies were conducted in Medline (Ovid), CINAHL (EBSCO), Web of Science, PubMed, and EMBASE. We performed the last search on March 14, 2025. The review followed the PRISMA guidelines for reporting [28]. The PRISMA checklist is provided as a related file. Restrictions were placed on language (English only) and years of publication (2004–2024) as we were only interested in recent evidence.

A copy of the search strategy for Medline is presented in Supplementary file 1.

Besides the main search, the lateral searching techniques were approached, which included analysing the reference lists of the included systematic reviews and the papers selected for inclusion after full-text reading [29].

### Inclusion and exclusion criteria

The relevant studies were chosen based on specific eligibility criteria using a two-part screening process. Two reviewers (DB and VK, both PhD students with medical qualifications) assessed articles by independently screening the titles, abstracts, and subsequent full text of potentially eligible papers. The same reviewers were responsible for the data extraction and the quality assessment process with the help from LHS. Any disagreements were resolved by discussion, and if not, a third researcher (DF or RTE) was consulted.

The “PICOS” framework has been chosen to shape the review question. The related systematic reviews on the relevant topics have informed the search strategy framework for this study [18]:

- Population – the roles of PAs, AAs and SCPs recognised in the UK.
- Intervention – incorporation of MAPs into the healthcare systems.
- Comparison – any healthcare professional to whom MAPs may be compared.
- Outcomes – any measure of impact on the defined healthcare productivity and quality indicators [30] and performance markers, patients’ perceptions of the roles, colleagues’ satisfaction with the roles, and the economic evaluation of the MAPs.
- Study design – any qualitative, quantitative, and mixed-method primary study designs that allowed the analysis of the impact on the quality of care and the patients’ and colleagues’ perceptions of the roles. Economic evaluation studies have also been considered for the budget implication analysis.

Studies were eligible for analysis if they met the following inclusion criteria listed in Table 1:

**Table 1:** Inclusion and exclusion criteria

Criteria	Inclusion	Exclusion
Population	The roles of PAs, AAs and SCPs recognised in the UK	All the other non-medical roles or a mixture of the roles presented in one result
Outcomes of Interest	The evidence of MAPs’ impact on quality of care, productivity, patient satisfaction, colleagues’ perception, or cost-effectiveness.	Other aspects of the MAPs’ profession (education, demographics, legislation, history, etc.).
Study design	Any primary study designs, qualitative, quantitative, or mixed methods, that analyse the outcomes of interest.	Published non-primary research studies, opinion pieces, audits, and non-peer-reviewed reports
Language	English	Languages other than English
Publication year	Between 2004–2024	Pre year 2004

Studies were not excluded based on the country, setting, or healthcare system to maintain the focus of the study on exploring the international experience of implementing MAPs and accumulating worldwide evidence to address the research questions.

### Data collection

A checklist has been utilised to extract general study characteristics, outcomes, findings, and conclusions from selected papers. Covidence systematic reviewing software was used to manage references, remove duplicates, extract data, create the final flow of included papers, and facilitate the work of the involved reviewers.

### Quality appraisal tools

The quality of the papers included in the research has been evaluated using the Joanna Briggs Institute (JBI) tool quality checklists, a validated tool for assessing primary research of quantitative and qualitative data in various fields [31] mixed methods studies, a different quality appraisal tool called the Mixed Methods Appraisal Tool was used, which has been tested for its efficiency and reliability [32]. Following common practice in recent reviews, the converted item-level responses were categorised as a percentage of “yes” responses and categorised as high, moderate, or low, which has been used in multiple published reviews employing JBI tools and aligns with JBI guidance to provide an overarching statement of study quality [33–35]. The CHEERS Checklist has been used to appraise the only cost-effectiveness study included in the final analysis [36].

### Data analysis

The narrative synthesis was chosen for a data analysis approach, which followed the four key elements according to Popay et al. [37]:

- Developing a theory of how the intervention works, why and for whom;
- Developing a preliminary synthesis of findings of included studies;
- Exploring relationships in the data;
- Assessing the robustness of the synthesis.

A meta-analysis was not conducted because the included studies varied in scope and outcomes. Instead, in the narrative synthesis, the studies were grouped based on the roles of interest (PAs, AAs and SCPs) and then further divided based on the defined outcomes.

### Database searches

**Fig. 1:**
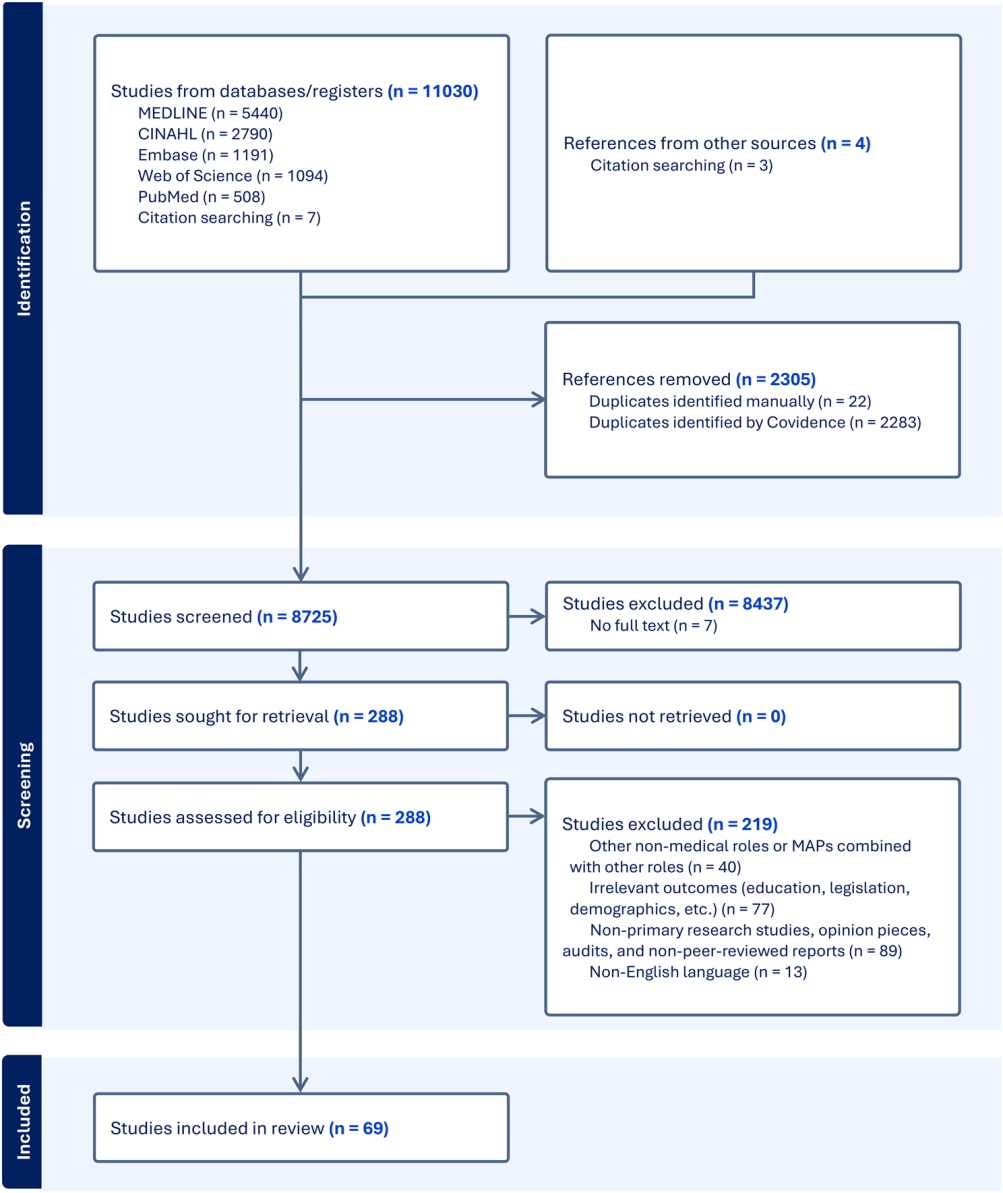
PRISMA flow chart

A total of 11,030 studies were identified for screening through the search strategy. After removing 2305 duplicates, 8725 papers were screened based on their titles and abstracts, out of which 8437 were excluded. The PRISMA flow chart in Fig. 1 illustrates the literature search and selection process and the reasons for study exclusion during full-text reading. During the full-text screening stage, a total of 288 studies were initially selected. Out of those, 219 studies were excluded as they did not meet the inclusion criteria. Specifically, 40 studies focused either on the other non-medical roles or combined MAPs with other professional groups, making it difficult to isolate the contribution of MAPs. A further 77 studies were excluded due to their focus on outcomes outside the scope of this review, such as educational pathways, legislative frameworks, or demographic trends. Another 89 publications were not considered eligible as they did not represent primary research, such as opinion pieces, audits, narrative reviews, and reports not subject to peer review. Finally, 13 studies were excluded due to being published in languages other than English. Data collection, quality appraisal, and analysis were conducted on a total of 69 papers. The evidence gathered from these studies is summarised below in four subsections. These subsections cover the characteristics of the studies, including their methodological quality and the synthesis of findings relevant to four defined outcomes of MAPs’ contribution.

## Results

This systematic review identified 69 studies on the impact of MAPs across various care settings. Most studies were from the USA (24), England (17), and Canada (12), with additions from other countries like the Netherlands, Germany, and Australia. The majority focused on PAs (61), while fewer studies examined SCPs (7) and AAs (1). MAPs were evaluated in different settings: five in tertiary care, 46 in secondary care, and the rest in primary or mixed healthcare environments. Findings indicate that PAs have the potential to generally improve some of the quality indicators, such as length of stay, readmission rates, and waiting times, though some exceptions exist. Evidence for SCPs shows comparable outcomes, while data on AAs are limited. Patient throughput and complication rates mostly stayed stable or improved with PAs, and mortality rates were unaffected, with two studies noting reductions. Healthcare staff reported generally high satisfaction with PAs, despite some confusion over roles. Patients were generally willing to be seen by PAs and were mostly satisfied with the care they received, especially if it shortened waiting times. Economic evaluations mostly favoured PAs and, to a lesser extent, SCPs, indicating lower care and operational costs. However, the studies’ low to moderate quality limits the strength of these conclusions.

### Study quality

In the supplementary files section (Supplementary file 2–8), the quality appraisal tables are presented. The papers identified within this systematic review vary in methodological quality, with the majority ranked as low or moderate quality according to the JBI tools. It was observed that the quantitative studies included in the research displayed some common methodological flaws, including the lack of adjustment in analysis for confounding variables, absence of information to assess the adequacy of participant selection, insufficient details regarding the baseline or demographic information of the participants under investigation, and a lack of specific information on the follow-up time required for outcomes to manifest. In qualitative studies, a recurring issue was the absence of information regarding the role of the researcher in the research and the philosophical foundation of the chosen methodology.

### Synthesis of findings on the impact of MAPs

In Table 2, a map of the included evidence shows the findings, which have been categorised according to the four research questions and defined outcomes. Each category has been analysed considering the population of interest, geographical setting, healthcare system, level of care presented, role representation, and the quality of papers.

**Table 2:** Map of a theoretical framework for primary studies

	Quality of care / Productivity							Colleagues perception	Patient perception		Economic evaluation
**Citation**	Length of stay	Readmissions	Mortality	Throughput of patients	Complications	Waiting times	Professionals’ capacity		Willingness to be seen by PAs	Patient satisfaction with provided care	
Odogwu S, 2024 [71]	**X**	**X**			**X**						
Ononye R, 2024 [62]				**X**							
Griffith C, 2023 [105]										**X**	
Misurka J, 2023 [61]				**X**				**X**			**X**
Gibson K, 2023 [51]	**X**										**X**
Burrows E, 2023 [90]								**X**			
Bendicksen D, 2022 [36]	**X**	**X**	**X**							**X**	
Sellers C, 2022 [78]								**X**			
Halvachizadeh S, 2022 [89]								**X**			
Korth M, 2022 [104]										**X**	
Malloy S, 2021 [63]						**X**					**X**
Tucker R, 2021 [60]				**X**							
Moore J, 2021 [47]	**X**	**X**				**X**					
De La Roche M, 2021 [43]	**X**					**X**					
Hains T, 2021 [29]		**X**		**X**		**X**					
Fung D, 2020 [48]	**X**	**X**	**X**								
Berkowitz O, 2020 [93]									**X**		
Halter M, 2020 [55]		**X**								**X**	
Smalley S, 2020 [76]								**X**			
Drennan V, 2020 [81]								**X**			
Senft J, 2019 [53]		**X**		**X**			**X**				**X**
Taylor F, 2019 [103]										**X**	
Drennan V, 2019 [98]										**X**	
Hains T, 2018 [88]								**X**			
Chatterjee S, 2018 [83]								**X**			
Hascall R, 2018 [70]							**X**				
Joyce P, 2018 [94]									**X**		
Timmermans M, 2017 [101]	**X**									**X**	
Timmermans M, 2017 [49]											**X**
Meijer K, 2017 [99]										**X**	
Hepp S, 2017 [66]							**X**	**X**		**X**	
Chao A, 2017 [67]							**X**				**X**
Drennan V, 2017 [73]								**X**			
Reed D, 2017 [58]				**X**		**X**					
Pavlik D, 2017 [54]		**X**									
Halter M, 2017 [102]										**X**	
Bowen S, 2016 [72]								**X**		**X**	
Resnick C, 2016 [68]					**X**		**X**				**X**
Althausen P, 2016 [40]	**X**				**X**	**X**					
Nabagiez J, 2016 [57]		**X**									**X**
Dies N, 2016 [59]				**X**			**X**				
Drennan V, 2015 [52]		**X**								**X**	
Decloe M, 2015 [41]	**X**		**X**			**X**					
Theunissen B, 2014 [50]	**X**					**X**					
Williams L, 2014 [84]								**X**			
Van Vught A, 2014 [75]								**X**			
Kumar R, 2013 [64]							**X**				
Nabagiez J, 2013 [56]		**X**									
White H, 2013 [80]								**X**			
Doan Q, 2013 [85]								**X**			
Doan Q, 2013 [91]									**X**		
Quick J, 2013 [77]								**X**			
Doan Q, 2012 [92]									**X**		
Kuilman L, 2012 [95]									**X**		
Ranzenbach E, 2012 [65]			**X**		**X**		**X**				
Berg G, 2012 [97]										**X**	
Farmer J, 2011 [100]										**X**	**X**
Drennan V, 2011 [74]								**X**			**X**
Kurti L, 2011 [82]								**X**			
Gifford A, 2011 [87]								**X**		**X**	
Singh S, 2011 [45]	**X**	**X**	**X**	**X**							
Bohm E, 2010 [69]							**X**	**X**		**X**	**X**
Hooker R, 2010 [96]									**X**		
Pereira C, 2010 [79]								**X**			**X**
Ducharme J, 2009 [42]	**X**					**X**					
Mains C, 2009 [39]	**X**		**X**								
Morgan P, 2008 [46]				**X**							
Kruk M, 2007 [107]											**X**
Rodi S, 2006 [44]	**X**									**X**	

### Impact on the quality of care and productivity

Quality measurement in healthcare has significantly advanced in recent decades and has garnered increasing interest among researchers, policymakers, and the public [30]. Currently, there is no standard method for measuring healthcare quality. As a result, researchers and policymakers are working to develop a systematic method for measuring and comparing the quality of care across different providers. Many countries now include a number of quality of care indicators in their overall health system performance reports to reflect the effectiveness of the system and the quality of care provided, encompassing patient experience, waiting times, complication rates, healthcare workforce capacity, and other relevant factors. Additionally, specific quality indicators, such as length of stay or readmission rates, may overlap with the concept of productivity in care, which assesses the amount of care provided relative to the funding available [38]. Productivity in healthcare, influenced by acute, non-acute, and staff productivity, is a key measure of healthcare performance, comparing the growth in output quantity to the growth in input quantity [39]. Despite the growing interest, productivity remains a difficult indicator to quantify and unify into a single definition or calculation method, causing numerous debates in studies that have aimed to identify the best way to measure and diagnose it [39]. Therefore, the overlap with the quality of care indicators is unavoidable in this section, but the list of outcomes has been determined by the identified evidence to accurately reflect the MAPs’ impact on them in a clear and transparent manner [40].

The seven most reported healthcare quality indicators are presented in Table 2 under the overarching term “Quality of care/productivity”. These quality indicators are:

1. Patients length of stay;
2. Readmission rates;
3. Complications;
4. Mortality;
5. Throughput of patients;
6. Waiting times;
7. Professionals’ capacity.

Most of the studies in this group are retrospective cohort studies with a “before/after” design. The objective was to compare different healthcare staff groups with and without MAPs and assess their impact on various healthcare quality and performance indicators. The complexity of the intervention, combined with challenges in study design, particularly with follow-up groups, and numerous confounding factors, significantly undermines the quality of most published papers, which are clearly of low to moderate quality (see Supplementary file 4).

#### Patient length of stay

Thirteen studies evaluated the length of stay (LOS) as an indicator of quality of care, which was one of the most commonly reported indicators among the included papers.

All studies focused on the impact of physician assistants (PAs) on LOS, while no papers examined the same question regarding AAs or SCPs. All of the studies have a retrospective observational design with a list of methodological limitations, including exposure measurement, strategies to deal with confounding factors, poor baseline information, and complicated follow-up.

Five studies conducted in the USA and Canada have shown that the inclusion of PAs in staff groups in secondary care units, such as traumatology, infectious diseases, or emergency departments, is associated with a lower LOS for patients compared to staff groups without PAs [41–45].

Two studies that evaluated the impact of PAs as a part of a new model of care on LOS illustrated contradictory evidence. One study found that the new Fast Track patient care model, which included input from PAs, led to a decrease in the length of stay (LOS) for patients [46]. However, another study showed that the new hospitalist-PA staff model was associated with longer LOS for inpatient care [47]. Another study that evaluated the PA-included and physicians-only models of care concluded the reduced number of visits for persons in the PA-included group by 16% [48].

Two other studies found that the care provided by PAs in the secondary care setting, particularly in the Intensive Care Unit (ICU) and Emergency Department (ED), resulted in longer average LOS for patients compared to the care provided by physicians [49, 50]. A final two studies in this group from the Netherlands illustrated that the PAs being introduced to the secondary care staff group were associated with not higher LOS for patients compared to the traditional staff model and, in the other case, the study showed a significantly better LOS compared to the staff group without a PA [51, 52].

The recent studies reported that the LOS for patients under the PA-physician model (74 h) was lower than for the physician-only model (83 h; *P* < 0.001) [38]. The same trend has been noted in another study where in the traumatology unit the PA-filled staff group registered lower LOS (M = 97.44, SD = 98.1) compared to the traditional one (M = 108.98, SD = 124) and the post-implementation group was calculated (M = 108.98, SD = 124; t[df] = -1.34, *P* = 0.18) [53].

#### Readmission rates

Eleven papers were identified that focused on evaluating the impact of MAPs on readmission rates at various levels of care. Most papers examined PAs, with only one exploring the influence of SCPs.

The studies conducted by Drennan and Singh showed no significant difference observed in the readmission rates of patients when comparing traditional primary care staff structures to those included in PAs [47, 54]. However, a study from Germany found that primary care institutions that employed PAs had a 3.5% lower rate of readmissions (*p* < 0.0463) [55].

Three of the four studies that were conducted in secondary care settings, specifically the ED, showed that the readmission rates were similar for the PAs and emergency physicians [49, 56, 57]. The remaining study was performed in the ICU department and came to the same findings as the studies above (PA 35.06; no PA 42.29, *p* = 0.46) [50].

Two studies explored the impact of the PAs on readmission rates in tertiary care and showed that the Physician Associate Home Care programme reduced the 30-day postoperative hospital readmission rate in patients who were discharged to home [58, 59]. It is worth noting that these studies had a moderate quality due to the limitations in the methodological quality of the sampling process.

The most recent study conducted in the secondary care setting found no significant difference in readmission rates between the PA-physician model of care and the physician-only model. (10.5% vs. 10.4%, *p* = 0.97), although the confounding factors in this study were not appropriately tackled [38].

The only study that was focused on SCPs confirmed no difference in readmission rates for care provided by SCPs compared to the medical surgical assistants [60].

#### Throughput of patients

Another widely mentioned outcome is average patient throughput, which is more of a productivity indicator than a quality indicator. Similar to other outcomes, the most evidence has been provided for the role of PAs compared to AAs and SCPs.

Three studies have examined the impact of PAs on patient throughput and hospitalisations in primary care, while two of them reported an increase in the volume of care provided when PAs were part of the team [61]. The other study has reported little to no change compared to teams without PAs [47, 55].

The other two papers were focused on exploring the influence of PAs on the same outcome in secondary care. One study found that PAs positively impacted the discharging routine compared to a team without a PA [62]. Another paper evaluated patient flow and electronic discharge efficiency and supported the hypothesis that a PA-included multidisciplinary team was not inferior to a traditional Medical Doctor-only team [63].

The study conducted in Australia assessed the impact on discharge rates of SCPs who assisted surgeries instead of medical surgical assistants and found no significant difference in the examined outcomes [60].

According to a recent study conducted by J. Misurka, PAs were found to increase the clinic volume by an average of 11.3 patient visits per day in the secondary care urology department, compared to the staff routine without the inclusion of PAs [64]. This study had a low quality due to the absence of a clearly described follow-up procedure for the participant cohorts and unclear strategies for addressing the confounding factors.

A recent study investigated how SCPs affect biopsy procedures and found that a well-supported, trained, and supervised surgical care practitioner can safely and effectively carry out TRUS systematic prostate biopsies, potentially enhancing access to prostate cancer diagnosis in developing countries [65].

#### MAPs impact on healthcare professional’ time and waiting times for patients

The electronic search identified nine studies that were focused on the impact of MAPs on patient waiting times. While 8 papers reflect the impact of the PAs, one study from Australia showed that the care provided by SCPs was no different regarding the waiting times rates and time in the operating room compared to the medical surgical assistants [60]. Only one study has been conducted on PAs in a primary care setting, and it illustrated that, considering that there were no presented strategies for dealing with confounding factors in the study, adding PAs to the team, decreased the waiting time for orthopaedic consultations at the Veterans Affairs Medical Center from 30 days to 10 days [61].

Three studies conducted in secondary care provided contradictory results. Two of them have shown that MAPs have a positive impact on waiting times in emergency care [44, 45]. In contrast, the other study found that the “door-to-door” time for patients in the ED was slightly longer for PAs compared to emergency physicians [49]. However, all these studies were low to moderate quality due to a lack of confounding factors, resistance and complications with the exposure measurement.

Another study showed that the implementation of the Fast Track - patient flow system structured with the PAs in the Netherlands led to a 69% decrease, equivalent to 41 min, in median waiting time [52]. The studies evaluating the impact of PAs in surgical, infection, and traumatology settings demonstrated that PAs’ involvement led to shorter procedure times and shorter waiting times for patients [42, 43, 66].

Nine papers were identified that focus on the potential MAPs’ impact on other professionals’ time and enhancing their capacity for more complex tasks. While eight papers explored the PAs’ influence, one study showed the positive impact on the consultants’ time provided by introducing the SCP into the surgical team in England [67].

One included study evaluated the impact of PAs on other healthcare professionals’ time in Germany’s primary care settings. This identified that primary care units that had integrated PAs into their teams had 8.2% fewer specialist consultations compared to other surgeries [55].

However, in secondary care, the collected evidence on PAs has shown their positive impact on reducing the time required for different surgical procedures, which impacts the capacity of other professionals in the multidisciplinary team involved in the surgery [68–70]. In other surgical settings, papers have focused on the surgeon’s involvement in the procedure, where PAs have been shown to positively impact surgeon capacity when implemented into the team [62, 71, 72]. One study focused on rounding interruptions in the ICU unit proved that the presence of PAs was associated with a 31.8% lower rate of interruptions that potentially decreased need for the physicians to switch between cognitive tasks [73].

#### Complications

Complications are another healthcare quality indicator that reflects patient safety, and the quality of care provided. For example, it could be post-operative complications, such as foreign bodies left during surgery, or more general issues, like hospital-acquired infections. Three papers were identified within this systematic review, and all explored the potential impact on such outcomes after the PAs were implemented in a multidisciplinary team (MDT). While two studies showed no significant difference in complication rates before and after the PAs were introduced to the unit [68, 71], one study confirmed a 4.67% decrease in postoperative complications in the trauma unit after the PAs joined the multidisciplinary team (*p* = 0.0034) [42].

One recent study that has evaluated the SCP role showed that laparoscopic cholecystectomies can be performed safely and effectively with very low complication rates and high day-case rates, which makes SCPs a useful addition to the established surgical staff group [74].

#### Mortality

Six papers that identified mortality rates as the measure of care quality, all focused on the PA role. Most of the studies confirmed that patients’ mortality rates for a certain unit didn’t significantly change after the introduction of the PAs to the department [38, 43, 47, 68].

However, two papers indicated that the implementation of PAs in the healthcare department was associated with an overall decrease in mortality rates for a certain department [41, 50].

### Healthcare professional colleagues’ perception of the MAP roles

Twenty-one studies were identified, reflecting the opinions on the roles of MAPs collected from different healthcare workforce groups with experience working with them in various healthcare settings. Seven of the 21 research papers were qualitative studies, and the remaining 14 were quantitative, including cross-sectional, cohort, and mixed-methods studies.

Five of the seven qualitative studies included reflected healthcare colleagues’ perceptions of physician assistants, with one paper per AA and one per SCP. It is essential to acknowledge that all these qualitative studies had low to moderate quality due to the unclear positioning of the researcher and their theoretical background or influence on the research, which is crucial for qualitative research. Additionally, many of these studies face challenges in obtaining evidence with ethical approval from the relevant regulatory bodies.

Three qualitative papers covered the healthcare staff’s perception of PAs in primary care settings. One of those studies analysed the professionals’ opinions about the PAs at the different stages of their implementation and showed that most concerns that were identified before the roles were implemented did not materialise and that the idea of introducing PAs to primary care received strong support from the physicians, who found the whole idea successful if realised with appropriate planning and preparation [75]. Two studies in England surveyed colleagues’ perceptions of PAs in primary care. The support for PAs was mainly managerial, reflecting new public management principles, while some professionals were not supportive [76]. Professionals’ attitudes varied based on their positions within the nursing and medical professions, indicating stratification within the profession, but PAs were perceived as able to do a high volume of work at the same time, requiring low levels of supervision [77].

A study from the Netherlands evaluated the most frequently stated motives for hiring PAs, which were to increase continuity and quality of care, and confirmed physicians’ general satisfaction with meeting these goals [78]. In South Africa, physicians were asked about their experiences with implementing PAs in their secondary care unit, and while most were satisfied with their professionalism, they expressed concerns about unclear guidelines and their inability to prescribe [79].

The only qualitative study that has covered the perceptions of healthcare staff towards the SCPs was conducted among members of surgical teams who have worked with SCPs and summarised that SCPs were identified as assistants and operators who enhanced patient care, helped maintain surgical services, and supported the training of junior doctors [80].

The most recent qualitative study on colleagues’ perception of MAPs was conducted in 2022 on AAs. The college tutors, clinical leads and trainee representatives confirmed that professional relationships between AAs and the anaesthetic team were overwhelmingly positive, and the general perception from all staff groups interviewed was that AAs’ inclusion resulted in fewer cancelled lists, more flexible rotas and better flow of staggered admissions and emergency lists [81].

The quality appraisal of the cross-sectional studies showed that most papers were of questionable quality due to unclear criteria for the sampling process and a lack of strategies for dealing with the confounding factors common for cross-sectional research. Regarding the mixed-method studies, they experienced difficulties explaining the adequate rationale for using that method and struggled with the appropriate interpretation of the qualitative and quantitative data, which is why most of them were of moderate quality.

The 14 quantitative and mixed method studies covered different perspectives of the professionals’ views on the MAP roles. For example, several studies that were focused on the PAs’ roles at the secondary care level and the colleagues’ perceptions of them concluded predominantly positive perceptions and satisfaction with the level of service produced [69, 72, 82–84].

The next studies evaluated general healthcare staff opinions on PAs without focusing on particular healthcare settings, and one of the studies from Australia showed that the majority of nurses and doctors who worked with the PAs believed that the PAs made a positive contribution to the healthcare team by increasing the capacity to meet patient needs, reducing on-call requirements for doctors, liaising with other clinical team members, streamlining procedures for efficient patient throughput and providing continuity during periods of doctor changeover [85]. A further two studies, from England and the United States of America (USA), evaluated the general satisfaction with PAs amongst doctors and showed that most physicians perceived certified PAs to be competent for specific skills and were generally satisfied with their role [86, 87].

One study reflected the physicians’ opinion on PAs in a tertiary care level unit and illustrated that physicians treating children in Paediatric Emergency Departments felt that PAs could appropriately contribute to the care of over half of the presented clinical conditions [88].

The study from the USA evaluated the physicians’ opinion on PAs through the prism of a potential increase in malpractice risk. Medical malpractice is defined as the failure of a healthcare provider to provide adequate treatment, which results in harm to a patient. Negligence is the main claim in most malpractice lawsuits, which includes delayed or incorrect diagnosis, improper drug treatment, failure to consult with other healthcare professionals, failure to obtain informed consent, and mismanagement of procedures [89]. As a result of the two-staged surveying, the percentage of American College of Emergency Physicians members who disagreed or strongly disagreed that PAs are more likely than physicians to commit medical malpractice was 71.6% in 2004 and 67.9% in 2009 [90].

A study conducted in Australia was the only one to focus on surgeons’ attitudes towards SCPs. The study found that out of 188 respondents, 64% were either “very supportive” or “supportive to some degree” of the role, while 20% were “undecided,” and 16% were “not supportive.” This indicates that many surgeons are in favour of the development of the SCP role in hospital care [91].

Recent studies have focused on how PAs are perceived in different healthcare settings. A Canadian study found that healthcare staff believes PAs are skilled professionals who reduce workload and contribute to residents’ education [64]. Meanwhile, a medical workforce survey conducted in Switzerland showed that PAs have a positive impact on daily routines and promote professional collaborations [92]. The most recent study conducted in Canada evaluated physicians’ opinions on PAs and found that between 82.6% and 94.0% of supervising physicians rated PAs as very good or excellent [93].

### Patients’ perception of the MAP roles

The included studies on the patients’ perceptions of the MAPs’ roles were divided into two broad areas: papers that reflect the willingness to be seen or receive care by MAPs and satisfaction with the care received from MAPs or the role in general.

#### Willingness to be seen by the MAPs

The “Willingness to be seen” papers have been conducted in various countries using the same approach. The study participants were offered the choice of whom they would like to be seen by in three different emergency scenarios: later by a doctor or sooner, but by a non-medical provider. The six studies were conducted in Canada, Israel, Ireland, the Netherlands, and Australia using the algorithm described above to evaluate the patients’ willingness to be seen and receive care by the PAs.

The papers in this group had several methodological issues, including unclear sampling and measurement of participants’ exposure to the intervention, as well as a lack of strategies to address confounding factors, which prevented them from being appraised as high-quality studies.

Based on the studies included, a vast majority of respondents (ranging from 91% to 99%) from different backgrounds and settings preferred to be seen by PAs and receive care from them if it saved them time [94–99].

#### Satisfaction with the received care and the general perception of the MAPs

The final analysis included 17 papers reflecting patient satisfaction with care from PAs in different healthcare settings. Most of the papers evaluated patients’ experiences with the PAs in secondary care settings and showed positive feedback from the patients towards the interaction with the PAs [46, 57, 69, 72, 90, 100, 101].

Another four papers identified by the electronic search strategy explored patient satisfaction with the care received from PAs in primary care settings. The English study compared patient satisfaction with the care received from the GPs and PAs and illustrated no difference in rates of patient satisfaction between the two groups of people who received care from different providers (1.00, 95% CI = 0.42 to 2.36, *P* = 0.99) [54]. A study from the Netherlands with the same research objective showed that Dutch patients appear to be as satisfied with the care received by PAs as GPs [102].

A study from Scotland confirmed that “patients were satisfied with the PAs” in a primary care setting [103] and the Dutch paper showed that the involvement of PAs was associated with better experiences of patients (β 0.49, 95% CI 0.22–0.76, *p* = 0.001) [104].

The cross-sectional studies of this group have met the methodological issues with the appropriate statistical analysis and the correct outcomes measurement, which made them appraised as papers of low to moderate quality.

Three qualitative papers are included for the final data synthesis, two of which are on patient satisfaction with the PAs in a primary care setting and one in a secondary one. The data from the Canadian study showed increased patient satisfaction with care after the PAs were implemented [75] while the study from England illustrated that most of the respondents, but not all, reported positive experiences and outcomes of their consultation underlying the confusion with the definition of the role and lack of understanding who is in front of them [105]. The same confusion was reported by the other study from England, where many participants, despite being satisfied with the received care in general, were recorded to misconceive PAs to be doctors, raising a potential risk of reduced trust in the PA relationship and negative implications for satisfaction with their PA encounter [106]. Moreover, all these qualitative studies struggled to present the main researcher’s theoretical background and its influence on the study, so the quality of these papers was not high.

The two most recent cross-sectional studies were conducted in the USA and focused on evaluating patient satisfaction with PAs in secondary care. One of the studies confirmed that the presence of a physician assistant in the clinic positively affected the 5-star rating for all the 16 Press Ganey patient satisfaction questions except one including overall satisfaction ([OR], 1.38; 95% CI, 1.03–1.85; *P* = 0.031), the likelihood of being recommended to others (OR, 1.57; 95% CI, 1.16–2.14; *P* = 0.004) and friendliness/courtesy (OR, 1.58; 95% CI, 1.17–2.13; *P* = 0.003) [107]. The study from 2023 compared patient satisfaction with dermatological care provided by dermatologists, PAs, and residents and concluded that patient satisfaction remained consistently high for all three groups, with no statistically significant differences observed between dermatologists and PAs and slightly lower scores for residents (*P* < 0.01) [108].

### Economic evaluation of the MAPs roles

The only economic evaluation identified in this review has been done in the Netherlands by Timmermans et al., 2017. The methodology details of this study describe the cost-utility analysis (CUA), which analyses QALY via the EuroQol (EQ-5D) questionnaire.

The study aimed to determine the cost-utility of the new PA-MD model of inpatient care compared to the traditional one, which consists of MDs only. Based on the data from 2292 participants, the study found no significant difference in QALY gain (+ 0.02, 95%CI − 0.01 to 0.05) when comparing the PA/ MD model with the MD model. Total costs per patient did not significantly differ between the groups (+€568, 95%CI −€254 to €1391, *p* = 0.175). Regarding the costs per item, a difference of €309 (€387.21 today) per patient (95%CI €29 to €588, *p* = 0.030) was found in favour of the MD model regarding length of stay. Personnel costs per patient for the provider primarily responsible for medical care on the ward were lower in the PA/MD model (−€11, 95%CI −€16 to −€6, *p* < 0.01).

In conclusion, the study indicated that the cost-effectiveness of wards managed by PAs in collaboration with MDs is comparable to the care provided by traditional house staffing. The inclusion of PAs may result in lower personnel costs, though it does not necessarily reduce overall healthcare expenses [51]. The CHEERS Checklist has been used to appraise the study, and its results are in the supplementary files (Supplementary file 2).

Few other studies have evaluated the financial implications of implementing PAs in secondary care units and have shown significantly lower costs for patients’ care and procedure expenses while having a PA in the team [70, 71, 82, 103].

One of the studies showed that the integration of the PAs into the surgery teams was a cost-neutral intervention as it did not differ much from having a traditional general practitioner (GP) surgical assist [72]. A study from Germany found no difference regarding the consultation rate of general practitioners and hospital costs while implementing the PAs in primary care but showed the 4.7% lower costs of total medication (*p* < 0.0001) in practices with the PAs as a part of staff [55].

The only study that evaluated the budget implications of the implementation of the PAs to tertiary level of care units was the USA research that showed savings of $977,500 for $25,300 over 2 years, or $39 in health care, in terms of hospital billing, for every $1 spent for a group filled with PAs as a part of the staff [59].

A single qualitative study that covered the perceptions on the PAs’ impact on the costs of care in primary care settings has been done in the UK and illustrated the views of the GPs and practice managers, who suggested, based on their experience and collected evidence, that employing a PA outweighed or at least balanced their costs and challenges with healthcare delivery [77].

Only one study covered the financial implications of the implementation of the SCPs to the surgical teams was conducted in Mozambique and confirmed that wider implementation of the te’cnicos de cirurgia (Mozambique’s analogue of the British SCP) led to a significant substantial difference in cost per surgery compare to the staff group consisted purely from the obstetricians or gynaecologists ($60.3 versus $144.1 per procedure), (£71.41 versus £170.53 in modern days) [109].

The two most recent studies that explored the impact on costs of care from using the PAs in secondary care settings came from the USA and Canada. One of the studies illustrated a reduced price of care per patient at $645.39 for an overall savings of $276,000, or 10.6%, on projected costs for patient care in the cohort group that included PAs [53]. Another study showed that PAs did not represent a financial burden on the urology practice plan at the Princess Margaret Cancer Centre in Canada, with a revenue gain of $16,800 [64].

## Discussion

This review highlights growing international interest in MAPs across various healthcare settings. Despite some concerns about study quality, the collected evidence indicates that PAs have the potential to enhance the quality and productivity of care by reducing length of stay, waiting times, and by maintaining or improving patient outcomes. The extracted data on SCPs showed comparable results, but evidence for AAs remains limited. The synthesised evidence also demonstrates that perceptions of MAPs are predominantly positive among colleagues and patients, though role clarity and scope of practice continue to cause confusion. Economic evaluation evidence suggests potential cost savings, particularly for PAs, although the quality of this evidence varies. These findings suggest that MAPs can strengthen healthcare provision, but their success depends on context. The discussion examines the key outcomes from the collected evidence regarding the impact of MAPs on defined outcome groups and the implications for policy and decision-makers in medical workforce planning.

### Principal findings

This novel systematic review identified 69 studies of MAPs working in various levels of care settings, locally and internationally. Most studies included in the analysis were from the USA (*n* = 24), followed by England (*n* = 17) and Canada (*n* = 12). However, the geographic range of the analysis was broadened by including papers from the Netherlands, Germany, Australia, Mozambique, Tanzania, Israel, Ireland, Switzerland, South Africa and Scotland.

The PA role was studied in most cases (61), while SCPs and AAs have each been the subject of only a few studies (7 and 1, respectively). Studies have evaluated the impact of all the MAP representatives in different healthcare settings. Among these studies, five have explored their impact in tertiary care settings, while 46 have focused on secondary care units. The remaining studies have examined MAPs’ impact in primary care and various mixed or general healthcare environments.

This systematic review examined various outcomes, primarily focusing on four areas: impact on the quality of care, colleagues’ perception, patient satisfaction, and cost-effectiveness of MAPs roles.

The outcome of the quality of care was divided into six different indicators that could have been impacted by MAPs while delivering healthcare services. The most common quality indicator was the patients’ LOS, which explored the PAs’ impact only and displayed the positive impact of PAs on the average patient’s LOS in various settings. However, only two papers suggested that the implementation of PAs in the ICU and ED was associated with a longer average LOS [49, 50].

Readmission rates are a key quality indicator assessed regarding MAPs’ impact. Ten of eleven papers were positive about PAs’ effects on patient readmission rates after joining the multidisciplinary team, while one paper on SCPs found no difference in readmission rates compared to medical surgical assistants [91].

Eight studies were optimistic regarding the PAs’ impact on the waiting times for patients after being added to the medical workforce team [42–45, 49, 52, 61, 66]. Meanwhile, one paper showed that the care provided by SCPs was no different regarding the waiting times rates and time in the operating room compared to the medical surgical assistants [91].

Another eight papers reported that PAs were effective in releasing healthcare professionals’ capacity [55, 62, 68–73]. Only one study explored this outcome for SCPs and confirmed that the implementation of that role positively impacted consultants’ time [67].

Only seven papers explored patient throughput as a productivity indicator for the evaluation of MAPs’ impact and all of them showed positive or neutral effects on the patients’ throughput provided by the teams filled with MAPs compared to the traditional ones [47, 55, 61–64, 91].

Five papers explored complications, as one of the indicators of healthcare quality [42, 65, 68, 71, 74]. While four papers showed no significant difference in complication rates before and after the PAs or SCPs were introduced to the unit, one study confirmed a 4.67% decrease in postoperative complications in the trauma unit after the PAs joined the multidisciplinary team.

Four of six papers focused on mortality rates showed that the introduction of PAs to a particular department did not significantly change patients’ mortality rates [38, 43, 47, 68]. However, the remaining two reported a significant decrease in mortality rates after the implementation of PAs [41, 50].

Healthcare colleagues expressed general satisfaction with the PA roles based on the number of cross-sectional, cohort, and mixed-methods studies [64, 69, 72, 82–88, 90–93] and seven qualitative studies [75–79, 81, 110]. Some papers raised concerns about the unclear scope of the roles and prescribing abilities. Still, both studies found that clinical staff were satisfied with the professionalism of these new roles and their contributions to unit performance.

According to the studies that evaluated patients’ willingness to be seen by a PA, most respondents (91% to 99%) from various backgrounds and settings preferred to receive care from PAs if it saved them time [94–99]. Additionally, 17 other papers showed that patients were generally satisfied with the care provided by PAs [38, 46, 54, 57, 69, 72, 75, 90, 100–103].

Twelve studies that explored the budget implications of having MAPs in healthcare staff teams illustrated significantly lower costs for patients’ care and procedure expenses while having a PA on the team [51, 53, 55, 59, 64, 66, 71, 72, 82, 103]. One qualitative study conducted amongst the primary care units showed that employing a PA outweighed or at least balanced their costs and challenges with healthcare delivery [77].

The only study that covered the SCPs’ impact on the costs of care was conducted in Mozambique and showed that wider implementation of the tecnicos de cirurgia (Mozambique’s equivalent of the British SCP) led to a significant substantial difference in cost per surgery compared to the staff group consisted purely from the obstetricians or gynaecologists [109].

After evaluating all the evidence gathered in this comprehensive systematic review, though the evidence ranges from moderate to low, it is reasonable to suggest that MAPs (mainly PAs and SCPs) may enhance productivity and quality of care when implemented correctly. Additionally, international data indicates that patients and colleagues perceive PAs, AAs, and SCPs positively, potentially benefiting the overall budget while ensuring safe and high-quality care.

While most of these papers have a low to moderate quality due to the complexity of the intervention, challenges with confounding factors, and difficulties in managing baseline data, they have the potential to spark discussions among health economists, policymakers, and clinicians about the impact of MAPs on healthcare delivery. They also provide evidence that MAPs have the potential to avoid adding additional financial pressure on personnel and healthcare budgets. Instead, the collected evidence suggests that they could save resources for healthcare budget holders while maintaining a decent level of care.

### Strengths and weaknesses

This review has systematically assessed the international body of literature that covers the MAPs roles that are rapidly expanding under the current UK healthcare policies. The four most relevant outcomes were selected to draw together the evidence to navigate the gaps in evidence on MAPs’ impact on quality of care, patients’ and colleagues’ perceptions and the cost-effectiveness of the roles. However, this excluded evidence on other non-medical roles and outcomes around them. Regarding the MAPs roles, it was excluded any studies, including education and development pathways of the roles, legal regulations and policy development surrounding the roles’ progress. All the papers where it was impossible to separate the input of PAs, AAs or SCPs were also excluded.

However, this systematic review presents a comprehensive analysis of recent literature regarding all three MAP roles and explores the international perspective on their deployment and development, which would provide valuable insights for the UK. Additionally, this research offers a novel perspective on the MAP concept, delivering an in-depth review of the roles and examining the international evidence.

This study includes robust study designs such as retrospective, cross-sectional, and qualitative studies. However, reports, opinion papers, non-systematic reviews, audits, and individual journal columns were excluded. The review suggests that the accuracy of the findings reported may have been influenced by the methods used in the studies. This is because not all studies used the same standardised or validated tool suitable for use within healthcare settings, such as analysing staff or patient surveys or counting costing analysis.

At some point, the search strategy was a source of limitations due to language, publishing year, or study design restrictions. Many opinion papers, as well as some reports and journal articles, were not included due to a lack of methodological background that was rigorous enough for the review criteria.

Another important limitation of the narrative synthesis is the definition of the MAP’s roles in different countries. For instance, in the USA, PAs are eligible to prescribe, which makes their input more visible and valuable for the teams, while in the UK, the role of a PA does not hold such power. However, recent messages from the GMC have assured support for the roles and their development, similar to how it is in other countries [111]. Such a comparison serves a scientific and practical purpose, and it could help evaluate the future of these roles in the UK healthcare setting. That is why it is important to raise the quality of the evidence on the effectiveness of the non-medical roles to address the identified gaps and inform the decision- and policy-makers with high-quality information that could comprehensively assist in the justification of healthcare workforce planning decisions.

Most of the included papers were from the USA and Canada, where health service organisations and the MAP roles may differ from those in other countries developing these roles. For example, in the USA, PAs can prescribe and order ionising radiation and are, as professionals, more experienced than in countries that have more recently embraced this role. Therefore, evidence from other healthcare systems identified within this systematic review does not guarantee the model’s success in the UK or elsewhere. It is important to emphasise that evidence from other countries cannot be transferred directly, as the structure and organisation of a nation’s healthcare system, along with cultural norms, economic development and levels of patient health literacy, are amongst the key factors in successful implementation of the roles or workforce structures that can significantly influence patient experience, quality of care or productivity of the healthcare system.

We could not conduct a thorough meta-analysis due to the varied nature of the data, interventions, and literature search methods used. Instead, we have opted for a narrative review, which may not be as precise, but we have provided a clear explanation for our synthesis and conclusions.

Several other aspects of MAPs deployment across different healthcare systems, such as legislation, historical context, demographics, optimal role integration patterns, educational strategies, and other critical factors for successful roles’ integration, have not been examined in this review and could serve as objectives for future research.

### Meaning of the study

This systematic review aims to update the recent evidence on the impact of MAPs on the quality of care, provide up-to-date information on healthcare staff and patient perceptions towards the roles, and explore their cost-effectiveness in various healthcare settings. Medical Associate Professions, especially PAs and AAs, recently became a hot topic in social media, political tribunes and healthcare environments. The background to this controversy is complex and has been discussed in a recent paper by McKee et al. [25]. However, the DHSC in the UK has recently reacted by introducing new legislation to regulate physician associates and anaesthesia associates [27].

Earlier this year, the DHSC conducted a consultation on the draft legislation and has now published its response. As per the new regulation, the profession will be regulated under the physician associate title, and the GMC will be the future regulator [112]. In response to a recent development, the British Medical Association (BMA) published an internal staff survey that reflected concerns of NHS staff towards MAPs roles, especially PAs [113]. These concerns were further highlighted by the Chief Editor of the British Medical Journal (BMJ) in an article that called for an “urgent review” of the future development of the roles [114]. In addition, the Chair of the Council of the Academy of Medical Royal Colleges expressed concerns about the naming of the roles and their future regulation under the GMC [115].

A significant milestone in the ongoing debate over MAPs was the release of the independent Leng Review, commissioned by the government [116]. The review was conducted amid rising concerns about safety, scope of practice, and the deployment of these roles within the NHS. It found no evidence that PAs or AAs endanger patient safety but identified ongoing issues with role clarity, implementation inconsistency, and the need for clearer boundaries. To tackle these issues, it is recommended that several actions be taken, such as renaming Physician Associates to Physician Assistants to better reflect their roles and enhance public understanding. Additional suggestions focused on strengthening regulation, promoting integration, and securing a sustainable, well-defined role for these professions within the future healthcare workforce.

This systematic review has identified gaps in the current global literature regarding MAPs impact on the healthcare delivery in various healthcare systems and aims to contribute to the existing evidence. It has provided evidence of current literature on the international experience of implementing MAP roles in healthcare staff groups and the level of acceptance they have received from the public and colleagues. Additionally, it presents possible cost implications that could arise from the wider implementation of these roles in the healthcare system to inform decision-makers, healthcare professionals, and the public about the MAP roles and their potential impact on the healthcare system.

## Conclusion

This systematic review illustrated that MAPs have the potential to complement traditional workforce configuration to support productivity and quality of care. There is evidence that they are acceptable to patients, and there is mixed evidence about their contribution in the eyes of clinical colleagues. A modest amount of data is available on PAs, and much less on SCPs and AAs. When assessed using standardised quality assessment tools, these papers were found to be of moderate to poor quality.

However, our rigorous and innovative systematic review suggests that incorporating PAs and SCPs into healthcare can provide a positive impact on the quality of care provided, more effectively manage the overall personnel costs and costs of care, and receive positive feedback from both patients and colleagues when appropriately implemented.

## Supplementary Information

Below is the link to the electronic supplementary material.

Supplementary Material 1

Supplementary Material 2

## Data Availability

Data sharing is not applicable to this article as no datasets were generated or analysed during the current study.

## Abbreviations

AAs: Anaesthesia Associates
BMA: British Medical Association
BMJ: British Medical Journal
DHSC: Department of Health and Social Care
ED: Emergency Department
EU: European Union
GMC: General Medical Council
GP: General Practitioner
HEE: Health Education England
ICU: Intensive Care Unit
JBI: Joanna Briggs Institute
LOS: Length of Stay
MAPs: Medical Associate Professionals
MD: Medical Doctor
MDT: Multidisciplinary team
NHS: National Health Service
PAs: Physician Associates
PRISMA: Preferred Reporting Items for Systematic Reviews and Meta-Analyses
SCP: Surgical Care Practitioner
UK: The United Kingdom
USA: United States of America
WHO: World Health Organisation
