# Supplementary material for: The impact of Medical Associate Professions (MAPs) on the productivity, quality of care, patient and healthcare workforce satisfaction, and budget implications in various healthcare settings: a systematic review
Source: BMC Health Serv Res. 2025 Nov 19;25:1491. doi: 10.1186/s12913-025-13626-4 (PMC12628565; doi:10.1186/s12913-025-13626-4)
Supplement: Supplementary file 1 — Supplementary Material 1 [file 12913_2025_13626_MOESM1_ESM.docx]

**EVIDENCE TABLE**

**QUANTITATIVE STUDIES**

| **First author and year** | **Country** | **Study design** | **Participants** | **Intervention** | **Comparator** | **Outcomes** | **Findings** | **Role/Level of care** |
| --- | --- | --- | --- | --- | --- | --- | --- | --- |
| Bendicksen D, 2022 | USA | Cohort study | n = 2,296 community hospital patients in 2015;  n = 1,828 community hospital patients in 2017;  Total = 4,124 | Implementing a PA-physician hospitalist model of care | Physician-only model of care | Length of stay (LOS), readmissions, discharge destination, patient satisfaction, in-hospital mortality | LOS for patients under the PA-physician model (74 hours) was lower than for the physician-only model (83 hours; P < .001). The PA-physician model team discharged more patients home than to another facility (PA-physician 77.6%, physician-only 74.3%; P = .03). Thirty-day readmissions were about 10% (P = .97) and patients reported respectful treatment in about 80% (P = .53) of cases in each cohort. | PAs (secondary care) |
| de la Roche M, 2021 | Canada | Cohort study | 9701 patients were seen by PA during the 87 days and 10776 patients were seen by others for the 96 days when the PA was not working. | Integrating a physician associate into the emergency department of the Belleville General Hospital. | Emergency department without a PA. | -LWBS (left without being seen);  -PIA (Provider Initial Assessment) time;  -LOS (length of stay). | The average daily percent LWBS in the PA group was 3.4%, versus 5.2% in the control group (P < .001). The 90th percentile PIA time for the PA group was 3.9 hours, whereas for the control group, it was 4.5 hours (P<.001). The average LOS for the PA group was 313.85 minutes, whereas in the control group, it was 348.91, with an average difference per patient of 35.06 minutes in favour of the PA group (P<.001). | PAs (secondary care) |
| Morgan P, 2008 | USA | Cohort study | The group of 1,762 adults who received care from the PAs and 111,184 control persons who received it from physicians. | Integration of PAs to the healthcare delivery at office-based visits (PA+ group). | Physician-only service. | The number of office-based visits reported for a person in 1 year | The risk ratio of .84 indicates that the number of visits per year is reduced by about 16% for persons in the PA+ group compared with persons in the physician-only group. | PAs (secondary care) |
| Nabagiez J, 2013 | USA | Cohort study | PAHC group totalled 340 patients and the control group of 361 patients at Staten Island University Hospital. | Introduction of PAHC (physician associate home care). | Control group without PAs. | Hospital readmissions | The overall readmission rate for the control group was 16% (59 patients) and 12% (42 patients) for the PAHC group, a 25% reduction in the rate of readmissions (P = .161). PAHC program reduced the 30-day postoperative hospital readmission rate by 25% in patients who were discharged to home. | PAs (tertiary care) |
| Nabagiez J, 2016 | USA | Cohort study | 1,709 adults underwent cardiovascular surgery, 1,185 of whom were discharged directly to their homes. The control group totalled 648 patients (55%) and the PAHC group, totalled 537 patients (45%). | Introduction of PAHC (physician associate home care). | Control group without PAs. | - Hospital readmissions;  - costs of care. | Total readmissions for the control group (n = 648) was 101 patients (16%) compared with the PAHC group (n = 537) total readmissions of 54 (10%), a 38% reduction in the rate of readmission (p = 0.0049). The average hospital bill per readmission was $39,100 for the control group and $56,600 for the PAHC group (p = 0.0547). Analysis demonstrated a savings of $977,500 at a cost of $25,300 over 2 years, or $39 in health care saved, in terms of hospital billing, for every $1 spent. | PAs (tertiary care) |
| Ranzenbach E, 2012 | USA | Cohort study | 956 patients at Enloe Medical Centre. Surgeon assistants assisted at 78% of all cases (n=748), while PAs assisted at 22% of all cases (n=208). | Implementation of PAs to the cardiac surgery teams. | Teams without PAs, but with surgeon assistants. | - OR time;  - Skin-to-skin incision time;  - Euroscore/Euromortality;  - Complication rates;  - Performance markers; | OR time was significantly less for PAs (4.09 hours ± 1.73; 95% CI 0.394) than for their surgeon counterparts (4.95 hours ± 2.0; 95% CI .211) (P =.002). Skin-to-skin incision time was also significantly shorter for PA-assisted cases (167.5 ± 82; 95% CI 11.23) than for surgeon assistants (205.9 ± 6.9; 95% CI 4.80) (P < .001). All other performance factors examined showed no significant difference between PA and surgeon assistants. | PAs (secondary care) |
| Reed D, 2017 | USA | Cohort study | From May to December 2012 – 10,600 encounters, from May to December 2014 – 13,885. | Implementation of the four new PAs added in 2013 to the CBOC orthopaedic staff. | CBOC orthopaedic staff structure before the project. | -waiting times;  -throughput of patients; | During the observation period of an added PA in orthopaedics in the CBOC, the wait time for an orthopaedic consultation at the VAMC decreased from 30 days (SD ± 25) to 10 days (SD ± 10). The number of orthopaedic encounters at the VAMC increased by 994 (11%) during the study period and overall encounters were up 31%. The Conroe CBOC also increased its orthopaedic encounter count by 10% during the same period of observation. A total of 3,909 orthopaedic encounters took place in the five CBOCs during this study period—a 383% increase over previous CBOC orthopaedic encounter volume. | PAs (primary care) |
| Resnick C, 2016 | USA | Cohort study | A total of 50 patients met the criteria for inclusion in the study. Each cohort contained 25 patients. | Incorporation of PAs into the practice. | Patients treated from August to November 2014 without the use of PAs (‘‘no PA group’’). | -costs;  - surgeons’ time;  -complications. | The total process time did not differ significantly between groups, but the average total procedure cost decreased by $75.08 after the introduction of PAs (P < .001). The time that the oral and maxillofacial surgeon was directly involved in the procedure decreased by an average of 19.2 minutes after the introduction of PAs (P < .001). No significant differences in postoperative complications were found. | PAs (secondary care) |
| Singh S, 2011 | USA | Cohort study | 9681 general medical (GM) hospitalizations between January 2005 and December 2006. | Inpatient care provided by a hospitalist-PA (H-PA) model. | Traditional resident-based model. | -Length of stay (LOS);  -charges;  -readmission within 7, 14, and 30 days;  -inpatient mortality | Inpatient care provided by H-PA teams was associated with a 6.73% longer LOS (P ¼ 0.005) but charges, risk of readmission at 7, 14, and 30 days and inpatient mortality were similar to resident-based teams. The increase in LOS was dependent on the time of admission of the patients. | PAs (primary care) |
| Timmermans M, 2017 | The Netherlands | Cohort study | Data on 2,307 patients from 34 hospital wards was included. PA/MD model - 1,021 patients in 17 wards MD-model - 1,286 patients in over 17 hospital wards. | Introduction of mixed model in which besides MDs PAs are employed (PA/MD model). | Traditional model in which only MDs are employed. | The primary outcome measure was patients’ LOS. Secondary outcomes concerned eleven indicators for the quality and safety of inpatient care and patients’ experiences with the provided care. | The involvement of PAs was not significantly associated with LOS (β 1.20, 95%CI 0.99–1.40, p = .062). None of the indicators for quality and safety of care were different between study arms. However, the involvement of PAs was associated with better experiences of patients (β 0.49, 95% CI 0.22–0.76, p = .001). | PAs (primary care) |
| Theunissen B, 2014 | The Netherlands | Cohort study | During the study period, 1,289 patients were treated before, and 1,393 after the introduction of FT. | Introduction of the new Fast Track patient flow system structured with physician assistants in the ED department. | Emergency department 3 months before the implementation of the FT system. | -waiting times;  -length of stay. | After the introduction of FT, it was observed a decrease of 12 min (13 %) in the median length of stay for the total group. The median waiting time decreased by 41 min (69 %). The group comprising patients with low to moderate urgency levels showed a median reduction of 12 min in length of stay, whereas the length of stay for urgent patients was reduced by 19 min. The waiting time for the low to moderate urgency patients decreased by 68 min, while the urgent patient group showed a reduction of 32 min. | PAs (secondary care) |
| Moore J, 2021 | USA | Cohort study | The analyses included 25,883 individual patient encounters managed by either a physician (10,678) or a PA (15,205). | Care provided by PAs at ED. | Care provided by physicians at ED. | -Length of stay;  -door-to-door time;  -72-hours readmission; | The mean length of stay (LOS) in this project was 120 (±96) minutes, with PAs demonstrating a slightly longer LOS (M = 126, ±96) compared with physicians (M = 120, ±96; P < .001). The mean time from patient entry into the ED until provider evaluation, or “door-to-doctor” time was 15.6 (±60) minutes among all providers. Physicians had slightly shorter door-to-doctor times (M = 11.4, ±108 minutes) compared with PAs (M = 16.2, ±66 minutes), P < .001. Although minor variances in metrics were appreciated, transfers, 72-hour returns, and death rates were all similar among provider types. | PAs (secondary care) |
| Hains T, 2021 | Australia | Cohort study | 956 surgical procedures undertaken at the Sunshine Coast University Private Hospital. | Intraoperative care provided by a non-medical surgical assistant. | Intraoperative care provided by medical surgical assistant. | -intraoperative time;  -time in operating room;  -ICU time;  -length of time;  -discharge rates;  -readmission rates. | Patient outcome assessment showed no statistically significant differences for surgical assistant types. | SCPs  (secondary care) |
| Pavlik D, 2017 | USA | Cohort study | During the 24-month study period, a total of 10,369 paediatric patients aged between 0 and 6 years were treated in the ED | Care provided by PAs | Care provided by emergency physicians. | -72-hour recidivism rates; | Recidivism rates for the 3 clinical groups were as follows: PA (6.8%), EP (8.0%), and PA & EP (9.3%) (P < 0.03). Patients admitted to the hospital on their return visits for the 3 clinical groups were as follows: PA (0.4%), EP (0.6%), and PA & EP (0.7%) (P = 0.2). Based on the outcome measure of 72-hour recidivism, PA management of pediatric patients 6 years or younger is similar to that of attending EPs. | PAs (secondary care) |
| Mains C, 2009 | USA | Cohort study | There were 15,297 adult patients  with trauma included in the analysis. | Addition of physician assistants to the core trauma panel. | Trauma service covered by in-house general surgery residents and by in-house trauma surgeons. | -Hospital LOS;  -mortality;  -ICU LOS. | The introduction of PAs to the core trauma panel (group 3 vs. group 2) decreased overall mortality (2.80% vs. 3.76%, p = 0.05), and reduced mean and median hospital LOS (4.32 days vs. 4.69 days, p = 0.05;  and 3.74 days vs. 3.88 days, p = 0.02, respectively). | PAs (secondary care) |
| Ononye R, 2024v | UK | Cohort study | 218 patients who underwent TRUS prostate systematic biopsy by a  surgical care practitioner between 2020 and 2022 | Addition of SCP to a surgery department. | Procedures done by doctors vs done by SCPs | - Complications  - Throughput  - Quality of care | A well-trained, supported, and supervised surgical care practitioner can safely and effectively perform TRUS  systematic prostate biopsies and may improve access to prostate cancer diagnosis in developing countries | SCPs (secondary care) |
| Fung D, 2020 | Canada | Cohort study | The matched case-control review of 268 patient ICU charts was conducted. No PA cohort (n=136) to a PA cohort (n=132). | Care received from PAs during the ICU stay. | Internal medicine team at ICU without the PAs. | -mortality;  -readmission rates;  -length of stay;  -hospital stay;  -admission notes management. | The use of a PA was associated with a trend towards lower mortality (42% vs 27%, p<0.07) and no difference in readmission rates or resource intensity. Post-PA patients had greater lengths of ICU and hospital stay (19 hrs, p<0.002; 2 days, p<0.002) and more complete admission notes (p<0.003). Adding a PA was associated with greater ICU and hospital length of stay, more complete admission notes and a trend towards improved mortality. | PAs (secondary care) |
| Malloy S, 2021 | USA | Cohort study | A total of 49 reduction mammaplasty procedures  were included in analyses: PA first-assist (n = 25) and resident  first-assist (n = 24). | Procedures first- assisted by a PA. | Procedures first-assisted by a surgical resident. | -Operative time;  -procedural charges. | Procedures first-assisted by a  surgical resident took a mean/median of 34 minutes longer and were $3750 more  expensive, respectively, than cases first-assisted by a PA (P < 0.01, both). | PAs (secondary care) |
| Senft J, 2019 | Germany | Cohort study | A total of 397 493 patients were treated in HCA practices, 463 730 patients attended non-HCA practices. | Introduction of healthcare assistants to primary care practices in Germany. | Practices that were not employing healthcare assistants. | -Rate of specialists consultations;  -rate of hospitalisations;  -rate of readmissions;  -follow-on drug prescriptions;  -costs of total medications. | Patients in HCA practices had an 8.2% lower rate of specialist consultations (p<0.0001), a 4.0% lower rate of hospitalisations (p<0.0001), a 3.5% lower rate of readmissions (p<0.0463), a 14.2% lower rate of follow-on drug prescriptions (p<0.0001), and 4.7% lower costs of total medication (p<0.0001). No difference was found regarding the consultation rate of general practitioners and hospital costs. | PAs (primary care) |
| Rodi S, 2006 | USA | Cohort study | 146 patients were given surveys  in the preintervention period  (completed = 87). In the postintervention  period 115 patients were given surveys  (completed =91). | Implementation of a fast-track unit (containing the PAs) specifically designed to meet the needs of low-acuity patients in our ED. | Postintervention group of patients. | -Length of stay;  -patient satisfaction. | Mean LOS was compared for the preintervention  and postintervention groups and was significantly  decreased (127–53 minutes; P < .001) after the initiation  of the fast track unit. satisfaction score was created by combining 4  satisfaction domains as described above. Each element  of this score, the score itself, and a question of overall satisfaction were all significantly improved in  the postintervention group. | PAs (secondary care) |
| Gibson K, 2023 | USA | Cohort study | The initial data set for the LOS analysis consisted of 682 patients: 253 in the preimplementation set and 429 in the postimplementation set. Time to VTE prophylaxis was evaluated for 1,610 patients: 443 in the preimplementation data set and 1,167 in the postimplementation data set. | Implementation of a PA to community-based academic Level 2 trauma facility for inpatient responsibilities. | Patients received care before the implementation of the PAs | -patient LOS;  -injury rates;  - Injury Severity Score (ISS);  -time to VTE prophylaxis;  -costs of care per patient. | The mean LOS between patients in the preimplementation group (M = 108.98, SD = 124) and the postimplementation group was calculated (M = 97.44, SD = 98.1; t[df] = -1.34, P = .18). A reduced price of care per patient was calculated at $645.39 for an overall savings of $276,000, or 10.6%, on projected costs for patient care in the postimplementation group compared with the preimplementation group. VTE prophylaxis between patients in the preimplementation group (M = 3,216.6795, SD = 3,970.1737) and the postimplementation group was (M = 2,608.7386, SD = 3,347.2281; t[df] = -3.087, P = .0021). | PAs (secondary care) |
| Hascall R, 2018 | USA | Cohort study | The family-centred rounds conducted on 2,657 patients. | Introduction of the PAs to the family-centred rounding at the ICU unit. | Rounding when PAs were not present. | -Rounding interruptions. | When a PA was not present during rounding, 178 of 1,137 (15.7%) encounters were interrupted. However, when a PA was present, 163 of 1,520 (10.7%) encounters were interrupted. This represents a 31.8% lower rate of interruptions when a PA was present. The presence of a PA during rounds was significantly associated (P < .001) with a 35.3% lower likelihood of an interruption occurring. | PAs (secondary care) |
| Berg G, 2012 | USA | Cross-sectional study | 251 level I and level II trauma patients. | Implementation of the PAs to trauma patient care. |  | -Perceived Interpersonal Care (PIC);  -Perceived Technical Care (PTC);  -Global Patients’ Satisfaction | Perceived Interpersonal Care had a strong direct effect on Perceived Technical Care (.886) and accounted for 78.5% of the variance. PIC (–.152) did not have a significant direct effect on Global Satisfaction. PTC (.834) had a significant direct effect on Global Satisfaction. PIC also had a significant indirect effect (.739) on Global Satisfaction through PTC. | PAs (secondary care) |
| Berkowitz O, 2020 | Israel | Cross-sectional study | A total of 7808 respondents began the online survey and 6639 finished yielding a completion rate of 85%. | Integration of the PAs to the healthcare system. | Compared to being treated by a doctor, but later. | -Willingness to be treated by a PA (patient perception). | The large majority of respondents (91.6%), were willing to see a PA if it would save time. Of those the majority (70%) were willing to see a PA under any of the time-saving options. One fifth (21.6%) were willing to see a PA only in certain time-saving scenarios. The remaining respondents (8.4%) were not willing to see a PA under any time-saving circumstance. | PAs |
| Burrows K, 2023 | Canada | Cross-sectional study | 93 physicians who have been supervising PAs | Employment of the PAs to the healthcare system. |  | -Experiences of supervising physicians working with PAs;  -demonstrate the scope of PA work activity;  -exploring physician satisfaction and their rating of PA competencies. | The percentage of supervising physicians, who rated the PAs as very good or excellent, ranged from 82.6% to 94.0%. | PAs |
| Chatterjee S, 2018 | USA | Cross-sectional study | 288 actively licensed physicians listed with the Kentucky Board of Medical Licensure (KBML). | Integration of PAs to the healthcare system. |  | - Do physicians perceive PAs to be competent in specific skills required of a health care provider?  -Are the physicians’ perceptions of PA competence in specific skills dependent on physicians’ experience with PAs? | An overwhelming majority of the physicians perceived certified PAs to be competent (n $ 161, $ 60%) for specific skills. The survey had internal consistency with Cronbach’s alpha ranging from 0.75 to 0.87 for competency domains. Effect size ranged from 0.44 to 0.98 for scores given by physicians with, versus without, experience with PAs. | PAs |
| Odogwu S, 2024 | UK | Cohort study | 170 patients were operated on | Integration of SCPs into the unit | SCP compared to the doctors | The primary outcome was any complication requiring intervention.  Secondary outcomes were minor complications, operative time, length of stay, conversion and readmission. | With a structured, supervised approach, SCPs  could be trained to take on more complex procedures and further strengthen the surgical workforce. | SCPs |
| Doan Q, 2012 | Canada | Cross-sectional study | 270 mothers accompanying their children to the BC Children’s Hospital, a tertiary care facility in Vancouver, BC. | Integration of PAs to the BC Children’s Hospital. | Compared to receiving care from a doctor, but later. | -Willingness to receive care from PAs | Regardless of the scenarios, 99% of participants opted for PAs under personal circumstances; 96% opted for PAs when the issue involved their children. The choice favouring the PA persisted, albeit at slightly lower proportions, as the difference in wait time between PAs and physicians decreased (85% and 67% for a difference in PA and physician wait time of 3 and 2 hours, respectively). | PAs |
| Doan Q, 2013 | Canada | Cross-sectional study | 152 physicians using the Pediatric Emergency Research Canada (PERC) network database. | Introduction of PAs to emergency paediatric care in Canada. |  | Three outcome measures were assessed: demographic information, familiarity with PAs, and PA clinical roles in the PED. | None of the 57 clinical categories achieved at least 85% agreement regarding PA management without direct physician involvement. Twenty-four clinical conditions had less equal 15% agreement that any PA involvement would be inappropriate. For the remaining 33 clinical conditions, more than 85% of respondents felt that PAs could appropriately manage but were divided between requiring direct and only indirect physician supervision. | PAs (tertiary care) |
| Doan Q, 2013 | Canada | Cross-sectional study | 273 adults seeking care for a child at British Columbia Children’s Hospital | Introduction of PAs to emergency paediatric care in Canada. |  | - willingness to have a child assessed and treated by a PA. | Regarding whether they would be willing to have their child receive treatment by a PA, 140 (51.3%) respondents answered, 107 (39.2%) said maybe, and 26 (9.2%) said never. Most respondents (64.1%) would choose to have their child seen by a PA instead of waiting for a physician if the waiting time reduction was at least 60 minutes. | PAs (tertiary care) |
| Gifford A, 2011 | USA | Cross-sectional study | 724 members of the American College of Emergency Physicians (ACEP). | Introduction of PAs into emergency care. |  | - emergency medicine physicians’ perceptions of PA malpractice risk, utilization, and patient satisfaction; | The percentage of physicians who disagreed or strongly disagreed that PAs are more likely than physicians to commit medical malpractice was 71.6% in 2004 and 67.9% in 2009. In 2004, 84.3% of the respondents, and in 2009, 81.8% of the physicians disagreed or strongly disagreed that PAs were more likely than physicians to be sued as a result of medical malpractice. Additionally, most of the respondents either agreed or strongly agreed that PAs in the ED decrease wait times for patients to be seen (85.2% in 2004; 91% in 2009). Likewise, 65.2% in 2004 and 75.4% in 2009 agreed or strongly agreed that PAs increase patient satisfaction. | PAs (secondary care) |
| Griffith C, 2023 | USA | Cross-sectional study | 12,386 outpatient visits with 25 dermatologists 6 PAs, and 26 dermatology residents at UTSW Medical Center. | Introduction of PAs into dermatology practice. |  | -Patient satisfaction with physician assistants. | Patient satisfaction remained consistently high for all three groups throughout the study period. Overall, there were no statistically significant differences observed between dermatologists and PAs. Scores were slightly lower for residents (P < 0.01). | PAs (secondary care) |
| Hains T, 2018 | Australia | Cross-sectional study | 445 surgeons registered with the Royal Australasian College of Surgeons (RACS). | Introduction of NMSA into the surgical practice in Australia. |  | - Surgeons’ attitudes and current practice regarding the role of the non-medical surgical assistant (analogue of SCP in the UK). | In the private sector in Australia, 188 respondents (64 per cent) were ‘very supportive’ or ‘supportive to some degree’ of the role, with 60 (20 per cent) ‘undecided’ and 48 (16 per cent) ‘not supportive’. | SCPs (secondary care) |
| Halvachizadeh S, 2022 | Switzerland | Cross-sectional study | 53 nurses and physicians who work full-time at a surgical ward in a Swiss reference centre. | Implementation of PAs to the surgical ward. |  | Administrative work, teamwork, improvement of workflow, and training of physicians have been assessed. | Participants reported a positive effect on the regular conduct of rounds (2.9, SD 1.1 points after 6 weeks and 3.5, SD 1.1 points after 12 weeks, p =0.05). A significant improvement in nurse-doctor collaboration has been reported (3.6, SD 1.0 and 4.2, SD 0.8, p=0.05). Nurses (n =28, 52.8%) reported that PAs provided a significant benefit effect on the surgical clinic (3.7, SD 1.0 points and 4.4, SD 0.8 points, p =0.043). Improved overall management of surgical cases was reported by the physicians (n =25, 47.2%) (4.8, SD 0.4 and 4.3, SD 0.6, p =0.046). | PAs |
| Kuilman L, 2012 | The Netherlands | Cross-sectional study | The study included 225 men and 225 women. | Integration of the PAs into outpatient care. | Compared to being seen by a doctor, but later. | -Willingness to be seen by a PA. | Surrogate patients chose the PA over the MD 96% to 98% of the time (depending on the scenario). No differences emerged when analysed by gender, age, or parenthood status. | PAs (secondary care) |
| Meijer K, 2017 | The Netherlands | Cross-sectional study | 99 patients seen by GPs and 115 patients seen by PAs. | Integration of the PAs to primary care. | Compared to received care from the GPs. | -patient satisfaction with care received from PAs. | This study found that Dutch patients appear to be as satisfied with the care received by PAs as GPs. | PAs (primary care) |
| Korth M, 2022 | USA | Cross-sectional study | 11,059 patients rated orthopaedic providers from a single institution’s outpatient orthopaedic surgery clinic. | Addition of the PAs to an outpatient orthopaedic clinic. |  | -patient satisfaction with PAs in outpatient orthopaedic surgery. | The presence of a physician assistant in the clinic positively affected the 5-star rating for all but 1 of the patient satisfaction questions examined, including overall satisfaction (odds ratio [OR], 1.38; 95% CI, 1.03-1.85; P=.031); the likelihood of being recommended to others (OR, 1.57; 95% CI, 1.16-2.14; P=.004); and friendliness/courtesy (OR, 1.58; 95% CI, 1.17-2.13; P=.003). | PAs (secondary care) |
| Williams L, 2014 | England | Cross-sectional study | 61 doctors completed the survey, representing 14 specialities or medical settings. | Implementation of the PAs to healthcare in the UK. |  | - Satisfaction of doctors with the role of physician associates. | In this survey of British doctors who currently work with PAs, doctors were generally satisfied with their role. Most doctors surveyed believed that the PAs possessed good clinical and communication skills and offered a beneficial continuity to practices and patients. | PAs |
| Joyce P, 2018 | Ireland | Cross-sectional study | 270 respondents took part in the study (67.5%) in two hospitals in Dublin. | Introduction of PAs to healthcare in Ireland. |  | -Willingness to be seen by a PA. | In total, 95% of the respondents chose to see a PA over a doctor based on the scenarios presented and a wait time of 30  minutes. | PAs |
| Hooker R, 2010 | Australia | Cross-sectional study | A total of 229 candidate patients unconditionally  participated (225 met the criteria). | Introduction of the PAs to a community health clinic in Australia. | Compared to being seen by a doctor, but later. | -Willingness to be seen by a PA. | All but two of the participants  (99%) selected to be treated by the PA regardless of the  scenario. When choices of time differences between a  doctor and a PA were reduced to 2 hours and 1 hour,  respectively, the preferential choice of seeing the PA  persisted. | PAs |
| Timmermans M, 2017 | The Netherlands | Cost-effectiveness analysis | 2292 patients were followed from admission  until 1 month after discharge. | Introduction of the PA/MD model of care to inpatient hospital care in the Netherlands. | Traditional MD-only model of care. | - All direct healthcare costs from the day of admission until 1month after discharge. Health outcomes concerned quality-adjusted life years (QALYs), which were measured with the EuroQol five dimensions questionnaire (EQ-5D). | It was found no significant difference for QALY gain (+0.02, 95%CI −0.01 to 0.05) when comparing the PA/ MD model with the MD model. Total costs per patient did not significantly differ between the groups (+€568, 95%CI −€254 to €1391, p=0.175). Regarding the costs per item, a difference of €309 per patient (95%CI €29 to €588, p=0.030) was found in favour of the MD model regarding length of stay. Personnel costs per patient for the provider who is primarily responsible for medical care on the ward were lower on the wards in the PA/MD model (−€11, 95%CI −€16 to −€6, p<0.01). | PAs (secondary care) |
| Althausen P, 2016 | USA | Case series | 1104 trauma patients with orthopaedic injuries. | PA involvement in Level ll trauma centre care |  | -Emergency room data;  -operating room data;  -complications rate;  -length of stay;  -postoperative DVT prophylaxis and prescriptions. | With PA involvement, trauma patients were seen 205 minutes faster (p = 0.006), total emergency room time decreased by 175 mins (p = 0.0001), and time to surgery improved by 360 mins (p > 0.03). Operating room parameters were minimally improved, but postoperative DVT prophylaxis increased by a mean of 6.73% (p = 0.0084), postoperative antibiotic administration increased by 2.88% (p = 0.0302), and there was a 4.67% decrease in postoperative complications (p = 0.0034). Average LOS decreased by 0.61 days (p = 0.27). | PAs (secondary care) |
| Decloe M, 2015 | Canada | Case-control study | Cases (n=3386) and controls (n=13,493). | Introduction of the PAs to the infectious diseases consult service (IDCS). | 2-year period before the introduction of the PAs. | -Time to consultation;  -length of stay;  -mortality rates. | Following the introduction of a PA to the IDCS, there was a decrease in time to consultation from 21.4 h to 14.3 h (P < 0.0001). LOS was significantly decreased among IDCS patients by 3.6 days more than that seen in matched hospital-wide controls (P=0.0001). Mortality did not significantly change after PA introduction in either cases or controls. | PAs (secondary care) |
| Kumar R, 2013 | England | Cohort study | 1558 OPD clinic appointments | Implementation of SCP to the upper gastrointestinal (UGI) consultant surgeon’s team. |  | -outpatient streamling;  -consultants’ time. | The general surgical care practitioner improves surgical outpatient streamlining and the delivery of elective surgical care. 330 of 1558 cases were managed by the SCP. The SCP therefore saved approximately 21% of outpatient appointments by more appropriate triaging and redirecting of referrals for major benign elective cases. | SCPs (secondary care) |
| Ducharme J, 2009 | Canada | Cohort study | 19,592 patient visits. PAs were on duty on 1076 visits with direct involvement with 396 patients. | The implementation of PAs in the six Ontario emergency departments. |  | -waiting times;  -LOS;  -patients who left without being seen (LWBS); | After adjustment  for hospitals, when a PA  was involved in patient care the odds of the patient  being seen within the benchmark wait time was  1.6 times greater than when the PA was not involved  (95% confidence interval [CI] 1.3–2.1, *p* < 0.05). When a PA was involved in patient care, the length of stay in the ED was 30.3% shorter than when a PA was not present (95% CI 21.6%–39%, *p* < 0.01). LWBS were 24.6% for PAs, the rate decreased from 6.5% to 4.9%. | PAs (secondary care) |
| Dies N, 2016 | USA | Cohort study | Group without PAs (n=126), group with PAs (n=848). | The implementation of the PAs in an academic surgical setting | Model of care without PAs | -discharge rates;  -quality of care;  -resident workload. | The teams with PAs had a 0.5% late discharge and 16% early discharge rate. Junior residents with a PA on the team spent fewer hours on the EMR. Residents reported PAs signifi cantly improved their rotation and quality care. | PAs (secondary care) |

**QUALITATIVE STUDIES EVIDENCE TABLE**

| **First author and year** | **Country** | **Study design** | **Participants** | **Intervention** | **Comparator** | **Outcomes** | **Findings** | **Role/Level of care** |
| --- | --- | --- | --- | --- | --- | --- | --- | --- |
| Drennan V, 2011 | England | Qualitative study (semi-structured interviews) | 13 GPs and three practice managers from 15 general practices employing PAs in five areas of England. | Recruiting PAs to work in GP practices in England, independent of any pilot schemes. |  | To evaluate the motivation of GPs to employ PAs and to understand the factors that sustained their employment. | **Motivation for employing PAs in general practice**: Many reasons were given for the initial decision to employ a PA. These included meeting government access time targets, increased patient demand, recruitment problems, and cost-effectiveness. One of the most frequently reported motives for starting to use PAs was the need to manage patient demand. A further factor was the difficulty experienced in recruiting doctors and nurse practitioners, particularly in practices in deprived urban areas and rural areas.  **The work of the PAs in general practice:** Participants described an incremental induction process to both accustom new PAs to English primary care and also assure GPs of their competence and safety. This involved the GP observing a PA’s consultations with patients with minor conditions before expanding the types of patients seen by PAs.  **The benefits of employing PAs:** PAs were perceived as able to do a high volume of work at the same time requiring low levels of supervision. Participants valued PAs’ ability to work within a medical framework, to manage uncertainty and to make decisions. PAs were also seen as acceptable to patients and this was illustrated by some participants citing examples of patients specifically asking to see the PA, although the reasons for this were unclear.  **The challenges and costs in employing PAs:** all participants reported that the advantages of employing a PA outweighed or at least balanced the costs and challenges. They were consistent in pointing to the same challenges: in particular, the lack of prescribing authority. The issue of acceptability of PAs to other professionals in the practice team, mostly nurses, was also raised. Most respondents noted that initially there was some form of ‘wariness’, bordering on resistance and hostility. | PAs (primary care – colleagues’ perception) |
| Halter M, 2017 | England | Qualitative study (semi-structured interviews) | 30 volunteer patients who had been consulted by physician associates for a same-day appointment in six general practices in England. | General practices employing physician associates. |  | To investigate the patients’ perspective on consulting with physician associates in general practice. | Some participants only consulted once with a physician associate and others more frequently. The conditions consulted for ranged from minor illnesses to those requiring immediate hospital admission. Understanding the role of the physician associate varied from ‘certain and correct’ to ‘uncertain’, to ‘certain and incorrect’, where the patient believed the physician associate to be a doctor. Most, but not all, reported positive experiences and outcomes of their consultation, with some choosing to consult the physician. Those with negative experiences described problems when the limits of the role were reached, requiring additional GP consultations or prescription delay. Trust and confidence in the physician associate was derived from trust in the NHS, the general practice and the individual physician associate. Willingness to consult a physician associate was contingent on the patient’s assessment of the severity or complexity of the problem and the desire for provider continuity. | PAs (primary care – patients’ perception) |
| Quick, 2013 | England | Qualitative study (semi-structured interviews) | All members of the general surgical team at one NHS trust in the West Midlands who had worked with a SCP for at least 6 months. | Implementing a SCP to the general surgical team. |  | To explore the experiences of members of the surgical team who work with a SCP. | Surgeons identified the provision of a knowledgeable, competent assistant and operator who enhanced patient care, helped maintain surgical services and supported the training of junior doctors. The professional, ethical and legal obligations of advanced perioperative practice were upheld. Interprofessional collaboration was improved, as was service provision. This further enhanced the patient experience. The traditional viewpoint that nurses who undertake tasks previously associated with medicine should be working to the standard of a doctor is challenged but requires further examination. | SCPs (secondary care – colleagues perception) |
| Van Vught A, 2014 | The Netherlands | Qualitative study (semi-structured interviews) | 55 specialists with the experience of working with PAs. | Incorporating of PAs into the healthcare system in the Netherlands. |  | To evaluate the experiences concerning outcomes such as quality of care and efficiency, acceptance of the PAs, and clinical competencies of the PAs. | With respect to the primary motives for employing a PA, the most frequently stated motive was to increase continuity and quality of care, followed by providing relief for the specialist’s workload, increase in efficiency of care, and substitution of the medical resident.  Regarding the outcomes of employing a PA, the most frequently mentioned were the increase in continuity of care, quality of care, efficiency of care, and workload relief for specialists. Efficiency of care was more often mentioned in the outcomes than in the motives. Some respondents reported a few problems concerning the acceptance of PAs. Furthermore, specialists demonstrated satisfaction  with the competencies of the PAs, according to expectations. Of 15 specialists, 11 described a “good” level of general clinical competencies and specialized clinical competencies; the remaining 4 described the same as “sufficient.” | PAs ( mixed level of care - colleagues’ perception) |
| Taylor F, 2019 | England | Qualitative study (semi-structured interviews) | 15 patients and patient representatives who had experienced a PA encounter across five hospitals in England. | Implementation of PAs to the acute hospital teams in England. |  | To understand patients’ satisfaction with PA acute hospital encounters through PA-patient communication experiences | **Feeling trust and confidence in the relationship:** trust in the expertise of the PA was a dominant consideration and the main criterion by which participants evaluated the quality of their encounter. Most participants perceived the PA that had attended them to have expertise and it was this expertise that formed the fulcrum of their confidence both in the PA and also the healthcare decisions resulting from the encounter.  **Sharing relevant and meaningful information:** PAs were perceived by many participants to be accessible and to encourage conversation. Provision of clear and informative explanations of the patient’s health status was an especially salient demonstration of the PA’s communication skills for many participants. Many participants expressed the view that provision of information about PAs and their role prior to a hospital encounter would be beneficial to prevent confusion and misunderstanding.  **Experiencing emotional care and support:** a frequently described aspect of the PA encounters was how participants felt that the PA had made them more at ease, modifying any feelings of fear or nervousness.  **Sharing discussion on illness management and treatment:** many participants described how their encounters with a PA had led to clarification or heightened understanding of the most appropriate way forward to treat or to manage their health condition.  The study identified four themes that collectively illustrate how study participants, who were generally satisfied with their PA acute hospital encounter, experienced PA-patient communication behaviours. | PAs (secondary care – patients’ perception) |
| Smalley S, 2020 | South Africa | Qualitative study (semi-structured interviews) | 24 physicians who directly supervised clinical associates at 17 district hospitals in the North West and Gauteng provinces. | The introduction of clinical associates at South African district hospitals in two provinces in 2012. |  | To determine the opinions of supervising physicians about the introduction of clinical associates at South African district hospitals in two provinces in 2012. | **Effects:** the most prominent comments relating to the positive theme of professionalism pertaining to the support provided to physicians by the clinical associates. Participating physicians described that their own work improved. The next most prominent positive theme included supervisor comments on the ability of the clinical associates to perform skills and procedures. A number of participants highlighted history taking, examinations, as well as making appropriate diagnoses as the skills they possess. Another positive theme identified was efficiencies in patient management highlighted by opinions of shortened patient waiting times, decreased patient complaints, more time spent with patients, and increased patient satisfaction.  **Concerns:** the most prominent concerns about the introduction of clinical associates pertained to administrative issues such as overtime and calls in the absence of clear guidelines of their roles. A further concern raised by participants was the inability of the clinical associates to prescribe medication without countersignatures by their supervising physician. Supervisors highlighted this as a limitation to the scope of the new cadre as well as a drawback in terms of providing greater efficiency. A third prominent negative theme was concern related to the supervision of clinical associates for their independence during practice and lack of training in some disciplines. | PAs (secondary care – colleagues’ perception) |
| Sellers C, 2022 | England | Qualitative study (semi-structured interviews) | 11 College Tutors or Clinical Leads, and 7 Trainee Representatives. | The deployment of Anaesthetic Associates as part of the Anaesthetic Team in the UK. |  | To investigate the deployment of Anaesthetic Associates in the UK in 2017;  -to explore the experience of working with AAs as part of the Anaesthetic Team;  -to assess their perceived impact on medical Anaesthetic training. | **Roles and relationships:** the experience of professional relationships between AAs and the anaesthetic team was overwhelmingly positive. All sites described a fraction of colleagues from both the Consultant and Trainee body who were sceptical of the role at the time of introduction to their departments, but all reported that the overwhelming majority of these had changed their opinion based on their experience.  **Impact on outcomes and efficiency:** it was reported that financial and clinical efficiency were not routinely measured, but the general perception from all staff groups interviewed was that AAs’ inclusion resulted in fewer cancelled lists, more flexible rotas and better flow of staggered admissions and emergency lists.  **Impact on medical anaesthetic training:** the overall experience of both College Tutors and Trainee Representatives in departments with established AA programmes was that there was no detriment to training opportunities and that there were some examples where AAs enhanced the training experience.  **Future developments:** key priorities for the future of the AA role were identified as securing regulation and the development of extended practices. Regulation was felt to be of central importance for the assurance of competency, supervision and responsibility, as well as in providing clarity of the role for colleagues, managers and patients. | AAs (secondary care – colleagues’ perception) |
| Drennan V, 2017 | England | Qualitative study (semi-structured interviews) | Macro and meso level semi-structured interview (n=25), micro level semi-structured interview (n=30). | The implementation of PAs to primary care in England. |  | To investigate the jurisdictional boundaries and relationships of a newly introduced occupational group into health care services, both at the system and workplace level. | The evidence from the macro and meso level of the health care system demonstrates the importance of state agency for the growth or otherwise of this new health profession, reflecting third type of interaction shaping professions. The arguments identified in a paper in support of PAs were largely managerial, reflecting the tenets of new public management and those against largely professional or occupational role protection. The degree of neutrality or degree of resistance to PAs by the participants from the nursing and medical professions varied according to their positions within the profession suggesting stratification, internal to the profession, shaped their perspectives. The evidence from the micro level presents the employment of PAs by GPs as clinician-managers, as a largely pragmatic, managerial response to medical and nursing shortages as opposed to active support for a new profession. However, GPs were split on their views about whether any advanced level clinical professional should be undertaking part of their medical work, reflecting a wider debate within medicine as to the nature and status of general practitioner work. | PAs (primary care – colleagues’ perception) |
| Bowen S, 2016 | Canada | Qualitative study | Phase 1 (n=16), phase 2 (n=24), phase 3 (n=74). | Implementation of PAs to primary care in Manitoba. |  | -patients’ perception;  -colleagues perception. | The concerns that were expressed during baseline interviews about the introduction of PAs (eg, community and patient acceptance) informed planning. Most concerns that were identified did not materialize. Supervising family physicians, site staff, and patients were enthusiastic about the introduction of PAs. There were a few challenges experienced at the site level (eg, front-desk scheduling), but they were perceived as manageable. Unanticipated challenges at the provincial level were identified (eg, diagnostic test ordering). Increased attachment and improved access—the goals of introducing PAs to primary care—were only some of the positive effects that were reported. | PAs (primary care – patients’ and colleagues’ perception) |

**MIXED METHODS STUDIES EVIDENCE TABLE**

| **First author and year** | **Country** | **Study design** | **Participants** | **Intervention** | **Comparator** | **Outcomes** | **Findings** | **Role/Level of care** |
| --- | --- | --- | --- | --- | --- | --- | --- | --- |
| Drennan V, 2019 | England | Mixed methods study | 43 PAs, 77 other health professionals, 28 managers, 28 patients and relatives. | Implementation of PAs to hospital care in England. |  | To investigate the deployment of physician associates (PAs);  the factors supporting and inhibiting their employment;  their contribution and impact on patients’ experience;  the outcomes and the organisation of services of PAs deployment. | **The deployment of PAs:** the PAs described themselves as belonging to the medical/surgical team, and their place in work rotas reflected this, with their main working hours being daytime on weekdays. Most PAs described their main work taking place on the ward or unit and this was evident from the work logs and observations. Only a small number of PAs undertook any work in outpatients or operating theatres and if so for a small percentage of their time.  **The contribution of PAs to patients’ experience:** patients and relatives reported very positive views of the PAs attending them. Particular aspects mentioned were: the PA’s constant presence on the ward meant they were easy to approach and PAs followed up items from the doctor’s ward round and spent time explaining decisions and management plans to the patients and relatives.  **The PAs’ impact on outcomes and the organisation of services:** The majority of doctors, nurses and managers described the contribution of PAs as positive. One of the most frequently reported impacts on the organisation was that PAs provided continuity of staffing in the medical/surgical team, that is, personal and team continuity. PAs were described and observed to undertake large amounts of non-patient facing clinical work for the medical/surgical team. The presence of a PA in the team was considered to release the doctors’ time to attend more complex patients and also to attend patients in outpatients and theatre. All consultants, registrars and managers reported the PAs to be safe with no serious incidents or patient complaints. | PAs (secondary care) |
| Halter M, 2020 | England | Mixed methods study | The records of 8816 patients attended by 6 PAs and 40 FY2 doctors; of these n=3197 had the primary outcome recorded (n=1129 physician associates, n=2068 doctor); 14 clinicians and managers and 6 patients or relatives for interview; 5 physician associates for observation. | The contribution of PAs to the processes and outcomes of emergency medicine consultations | The contribution of foundation year two doctors-in-training. | The primary outcome: unplanned re-attendance at the same emergency department within 7 days. Secondary outcomes: consultation processes, clinical adequacy of care, and staff and patient experience. | Re-attendances within 7 days (n=194 (6.1%)) showed no difference between physician associates and foundation year two doctors-in-training (OR 0.87, 95% CI 0.61 to 1.24, p=0.437). If seen by a physician associate, patients were more likely to receive an X-ray investigation (OR 2.10, 95% CI 1.72 to 4.24), p<0.001), after adjustment for patient characteristics, triage severity of condition and statistically significant clinician intraclass correlation. Clinical reviewers found almost all patients’ charts clinically adequate. Physician associates were evaluated as assessing patients in a similar way to foundation year two doctors-in-training and providing continuity in the team. Patients were positive about the care they had received from a physician associate but had poor understanding of the role. | PAs (secondary care) |
| Hepp S, 2017 | Canada | Mixed methods study | Semi-structured interviews (n = 38), health care provider (n = 28), patient surveys (n = 47). | The implementation of 1 PA position in an upper-extremity surgical program in Alberta, Canada. |  | -patients seen;  -colleagues’ and patients’ perception;  -preparation time, surgeon time and postsurgery time. | Preoperatively, the PA prioritizes patient referrals for surgery and redirects patients to alternative care. In the second year with the PA in place, there was an increase in total new patients seen (113%). Postoperatively, the PA attended rounds on 5 surgeons’ patients and handled follow-up care activities. Health care providers and patients reported that the PA provided excellent care. Findings from the operating room showed that the preparation time was greater than expected (38.6%), whereas the surgeon time (20.6%) and postsurgery time (37.2%) was less than expected. | PAs (secondary care) |
| Kruk M, 2007 | Mozambique | Mixed methods study | Interviews and a review of budgets, annual expenditure reports, enrolment registers, and accounting statements from training institutions. | The implementation of tecnicos de cirurgia (British analogue of SCPs) in hospital care in Mozambique |  | Cost per major obstetric surgical procedure over 30 years in 2006 US dollars. | The 30-year cost per major obstetric surgery was $38.9 for te´cnicos de cirurgia and $144.1 for surgeons and obstetrician/ gynaecologists. Doubling the salaries of te´cnicos de cirurgia resulted in a smaller but still substantial difference in cost per surgery between the groups ($60.3 versus $144.1 per procedure). One-way sensitivity analysis to test the impact of varying other inputs did not substantially change the magnitude of the cost advantage of tecnicos de cirurgia. | SCPs (secondary care) |
| Pereira C, 2010 | Mozambique and Tanzania | Mixed methods study | Cohort study (n=958, n=1113), operations analysis (n=12,178), semi-structured interviews (n=71), prospective study (n=1,134) | Implementation of AMOs (PAs) and TCs (SPCs) to the healthcare in Tanzania and Mozambique. |  | -costs;  -colleagues’ perception | There were no clinically significant differences in the outcomes of 2,071 consecutive caesarean sections performed in the two groups (TCs and specialists) at Maputo Central Hospital. The cost-effectiveness of TCs performing obstetric surgery was three times more favourable for TCs than for medical officers. Health staffs recognize with satisfaction that TCs alleviate the burden for medical officers resulting in a reduction of the need for patient referrals with cost reduction for patients. | PAs (secondary care) |
| Misurka J, 2023 | Canada | Cohort study | Retrospective analysis, survey (n=41), costs analysis. | Implementation of PAs to the urology department in Princess Margaret Cancer Centre, Canada. |  | -throughput of patients;  -costs;  -collegues’ perception. | On average, PAs increased clinic volume by 11.3 patient visits per day. Furthermore, they individually care for an average of 24 patients per day. PAs did not represent a financial burden on the urology practice plan (revenue gain of $ 16,800). The questionnaire demonstrated that PAs were capable healthcare professionals, who decreased workload and contributed to resident/fellow education. | PAs (secondary care) |
| Chao A, 2017 | USA | Cohort study | Retrospective analysis. A total of 4141 clinic encounters and 1356 surgical cases were reviewed. | A 1-year period after the addition of a PA into breast surgery practice. The practice model was a one-to-one pairing of a plastic surgeon and a PA. | A 1-year period before the implementation of the PAs. | -value units;  -opertaive time;  -surgeons time;  -costs. | A total of 4141 clinic encounters and 1356 surgical cases were reviewed. After the addition of PAs, there was a significant increase in relative value units (1057 vs 1323 per month per surgeon, P < 0.001). Operative times were similar with and without PAs (P = 0.45). However, clinic encounter times for surgeons were shorter for all visit types when patients were first seen by a PA before the surgeon: global follow-up (P = 0.03), other follow-up (P = 0.002), consultation (P = 0.76), and preoperative (P = 0.02), translating to 9 additional patients seen per day. Charges (P = 0.001) and payments (P = 0.007) also increased, which offset the cost of using a PA. However, the financial contribution from PA involvement as first assistant in surgery was limited (5.2%). The peak effect of PAs was observed between the third and fourth quarters. | PAs (secondary care) |
| Tucker R, 2021 | England | Mixed methods study | Survey (n=15), | The implementation of PA/MD at T&O inpatient service during the COVID-19 pandemic. | MD-only model of care in general surgical team. | -patient safety;  -continuity of care;  -patient flow;  -medical ward support.  -VTE assessment;  -electronic discharge efficiency. | Sixty-five per cent of responses indicated an improvement compared with pre-COVID conditions and 35% indicated care was the same. The electronic discharge notification audit showed an 89% completion rate for orthopaedics compared with 73% for general surgery. Venous thromboembolism assessment compliance was better compared with general surgery. Overall, the study supports the hypothesis that a PA/MD model of care is non-inferior to a MD-only model of care and was effective. | PAs (secondary care) |
| White H, 2013 | England | Mixed methods study | Survey 1 (n=19), survey 2 (n=9), interviews (n=11), survey 3 (n=3). | The implementation of PAs in the intensive care unit. |  | -colleagues’ perception towards PAs through a 10-month period. | Initially, there was a large discrepancy between expectations and the capabilities of the PAs. Shortly after starting, there was friction arising from PAs being untrained in PICU activities, and the facts that they would take training opportunities from other staff and that their remuneration was disproportionate to their usefulness. At five months, all those interviewed stressed the positive impact of PAs on patient care and the running of the unit. Staff had found that the PAs had integrated well and there was little evidence of earlier frictions. When surveyed at 10 months, PAs were undertaking most PICU procedures, albeit with some supervision. The study shows that PAs can be a valuable addition to the medical workforce, but that predictable problems can mar their introduction. Solutions are suggested for other units intending to follow this model. | PAs (secondary care) |
| Kurti L, 2011 | Australia | Mixed methods study | Pre- and post-surveys of Pilot site staff (n = 59 pre-Pilot, n = 40 post-Pilot), patient surveys (n = 99), 180 interviews with 106 individuals across sites, 14 stakeholders interviews. | Recruitment of five PAs to different primary and secondary care units in Queensland for 12 months. |  | -colleagues’ perception;  -quality of care;  -safety. | The evaluation found that the PAs provided quality, safe clinical care under the supervision of local medical officers. The majority of nurses and doctors who worked with the PAs believed that the PAs made a positive contribution to the health care team by increasing the capacity to meet patient needs; reducing on-call requirements for doctors; liaising with other clinical team members; streamlining procedures for efficient patient throughput; and providing continuity during periods of doctor changeover. The Pilot demonstrated that a delegated PA role can provide safe, quality healthcare by augmenting an established healthcare team. The PA role has the potential to benefit the community by increasing the capacity of the health care system and to improve recruitment and retention by providing an additional professional pathway. | PAs (primary and secondary care) |
| Bohm E, 2010 | Canada | Cross-sectional study | Arthroplasty cases (n=1409), surgeons survey (n=4), nurses survey (n=12), ward nurses survey (n=22), residents survey (n=6), patients survey (n=25), cost analysis (m=402). | The implementation of PAs to Canadian arthroplasty programme. |  | -time savings;  -colleagues perception;  -patients’ perception;  -costs;  -throughput of patients;  -waiting times. | PAs “saved” their supervising physician about 204 hours per year; this time can be used for other clinical, administrative or research duties. Physician assistants are regarded as important members of the health care team by surgeons, nurses, orthopedic residents and patients. When we compared the billing costs with those that would have been generated by the use of GP surgical assists, PAs were essentially cost neutral. Furthermore, they potentially freed GPs from the operating room to spend more time delivering primary care. We found that use of the double operating room model facilitated by PAs increased the surgical throughput of primary hip and knee replacements by 42%, and median wait times decreased from 44 weeks to 30 weeks compared with the preceding year. | PAs (secondary care) |
| Drennan V, 2015 | England | Cohort study | Patients’ clinical records (n=2086), survey (n=1020). | Implementation of PAs to the primary care in England. | Care received from a GP. | -reconsultation within 14 days;  -patients’ satisfaction. | There were no significant differences in the rates of re-consultation (rate ratio 1.24, 95% confidence interval [CI] = 0.86 to 1.79, P = 0.25). There were no differences in rates of diagnostic tests ordered (1.08, 95% CI = 0.89 to 1.30, P = 0.44), referrals (0.95, 95% CI = 0.63 to 1.43, P = 0.80), prescriptions issued (1.16, 95% CI = 0.87 to 1.53, P = 0.31), or patient satisfaction (1.00, 95% CI = 0.42 to 2.36, P = 0.99). Records of initial consultations of 79.2% (n = 145) of PAs and 48.3% (n = 99) of GPs were judged appropriate by independent GPs (P<0.001). The adjusted average PA consultation was 5.8 minutes longer than the GP consultation (95% CI = 2.46 to 7.1; P<0.001). | PAs (primary care) |
| Drennan V, 2020 | England | Mixed methods | 36 medical directors, consultants, junior doctors, nurses and managers, 198 documents. | A two-year programme employing 27 American physician associates in English hospitals. |  | -colleagues’ perception; | Over time, the experienced physician associates became viewed as a positive asset to medical and surgical teams. Their positive contribution was described as bringing continuity to the medical/surgical team which benefited patients, consultants, doctors-in-training, nurses and the overall efficiency of the service. Many reported the lack of physician associates regulation with attendant legislated authority to prescribe medicines and order ionising radiation was a hindrance in their deployment and employment. | PAs (secondary care) |
| Farmer J, 2011 | Scotland | Mixed methods | 15 USA-trained PAs, medical supervisors and team members, 20 patients, four NHS senior managers and three trade union representatives. | The implementation of PAs to the NHS Scotland. |  | -patinet satisfaction;  -scope of practice;  -costs. | Two minor patient safety issues arose. Patients were satisfied with PAs. Scope of practice did not replicate US working. Inability to prescribe was a hindrance. PAs tended to have longer consultations, but provided continuity and an educational resource. They were assessed to be mid-level practitioners approximating to nurse practitioner or generalist doctor. Valued features were generalism, medical background, confidence differential diagnosis and communication. Interviewees suggested PAs could fulfil roles currently filled by medical staff, potentially saving resources. | PAs (primary and secondary care) |
