# Supplementary material for: The impact of Medical Associate Professions (MAPs) on the productivity, quality of care, patient and healthcare workforce satisfaction, and budget implications in various healthcare settings: a systematic review
Source: BMC Health Serv Res. 2025 Nov 19;25:1491. doi: 10.1186/s12913-025-13626-4 (PMC12628565; doi:10.1186/s12913-025-13626-4)
Supplement: Supplementary file 2 — Supplementary Material 2 [file 12913_2025_13626_MOESM2_ESM.docx]

# Supplementary files

## Supplementary file 1. Medline search strategy


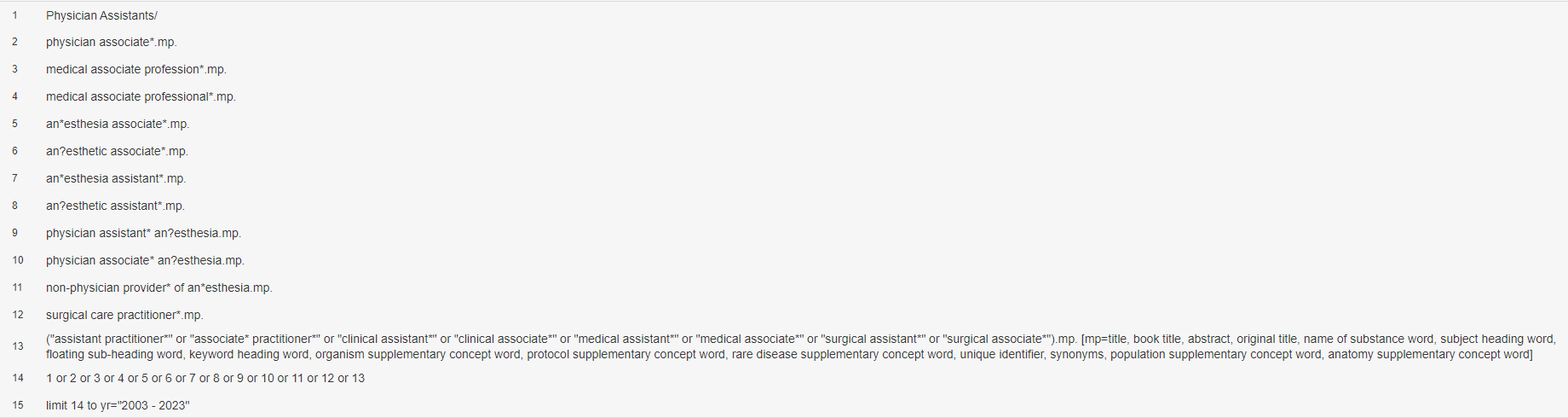


## Supplementary file 2. CHEERS 2022 Checklist

| **Topic** | **No.** | **Item** | **Location where item is reported** |
| --- | --- | --- | --- |
| **Title** |  |  |  |
|  | 1 | Identify the study as an economic evaluation and specify the interventions being compared. | Page 1, line 1 to 2 |
| **Abstract** |  |  |  |
|  | 2 | Provide a structured summary that highlights context, key methods, results, and alternative analyses. | Page 1, line 10 to 34 |
| **Introduction** |  |  |  |
| **Background and objectives** | 3 | Give the context for the study, the study question, and its practical relevance for decision making in policy or practice. | Page 1, line 36 to Page 2, line 20 |
| **Methods** |  |  |  |
| **Health economic analysis plan** | 4 | Indicate whether a health economic analysis plan was developed and where available. | Page 3, line 42 to 79 |
| **Study population** | 5 | Describe characteristics of the study population (such as age range, demographics, socioeconomic, or clinical characteristics). | Page 2, line 68 to 73 |
| **Setting and location** | 6 | Provide relevant contextual information that may influence findings. | Page 1, line 18 |
| **Comparators** | 7 | Describe the interventions or strategies being compared and why chosen. | Page 2, line 29 to 40 |
| **Perspective** | 8 | State the perspective(s) adopted by the study and why chosen. | Page 2, line 65 to 67 |
| **Time horizon** | 9 | State the time horizon for the study and why appropriate. | Page 3, line 82 to 85 |
| **Discount rate** | 10 | Report the discount rate(s) and reason chosen. | Not reported |
| **Selection of outcomes** | 11 | Describe what outcomes were used as the measure(s) of benefit(s) and harm(s). | Page 2, line 68 to Page 3, line 25 |
| **Measurement of outcomes** | 12 | Describe how outcomes used to capture benefit(s) and harm(s) were measured. | Page 2, line 17 to 20 |
| **Valuation of outcomes** | 13 | Describe the population and methods used to measure and value outcomes. | Page 2, line 68 to Page 3, line 25 |
| **Measurement and valuation of resources and costs** | 14 | Describe how costs were valued. | Page 3, line 18 to 21 |
| **Currency, price date, and conversion** | 15 | Report the dates of the estimated resource quantities and unit costs, plus the currency and year of conversion. | Page 3, line 21 to 24 |
| **Rationale and description of model** | 16 | If modelling is used, describe in detail and why used. Report if the model is publicly available and where it can be accessed. | Page 3, line 49 to 60 |
| **Analytics and assumptions** | 17 | Describe any methods for analysing or statistically transforming data, any extrapolation methods, and approaches for validating any model used. | Not reported |
| **Characterising heterogeneity** | 18 | Describe any methods used for estimating how the results of the study vary for subgroups. | Page 3, line 67 to 75 |
| **Characterising distributional effects** | 19 | Describe how impacts are distributed across different individuals or adjustments made to reflect priority populations. | Not reported |
| **Characterising uncertainty** | 20 | Describe methods to characterise any sources of uncertainty in the analysis. | Page 3, line 62 to 65 |
| **Approach to engagement with patients and others affected by the study** | 21 | Describe any approaches to engage patients or service recipients, the general public, communities, or stakeholders (such as clinicians or payers) in the design of the study. | Not reported |
| **Results** |  |  |  |
| **Study parameters** | 22 | Report all analytic inputs (such as values, ranges, references) including uncertainty or distributional assumptions. | Page 3, line 98 to Page 4, line 54 |
| **Summary of main results** | 23 | Report the mean values for the main categories of costs and outcomes of interest and summarise them in the most appropriate overall measure. | Page 4, line 54 to 60 |
| **Effect of uncertainty** | 24 | Describe how uncertainty about analytic judgments, inputs, or projections affect findings. Report the effect of choice of discount rate and time horizon, if applicable. | Page 4, line 33 to 36 |
| **Effect of engagement with patients and others affected by the study** | 25 | Report on any difference patient/service recipient, general public, community, or stakeholder involvement made to the approach or findings of the study | Not reported |
| **Discussion** |  |  |  |
| **Study findings, limitations, generalisability, and current knowledge** | 26 | Report key findings, limitations, ethical or equity considerations not captured, and how these could affect patients, policy, or practice. | Page 3, line 78 to 85, Page 9, line 7 to 28, Page 9, line 29 to 34 |
| **Other relevant information** |  |  |  |
| **Source of funding** | 27 | Describe how the study was funded and any role of the funder in the identification, design, conduct, and reporting of the analysis | Page 9, line 80 to 81 |
| **Conflicts of interest** | 28 | Report authors conflicts of interest according to journal or International Committee of Medical Journal Editors requirements. | Page 9, line 82 |

## Supplementary file 3. Quality assessment of the cohort studies

**JBI critical appraisal checklist for cohort studies**

| **Study** | **JBI Appraisal items** | | | | | | | | | | | **Score** |
| --- | --- | --- | --- | --- | --- | --- | --- | --- | --- | --- | --- | --- |
|  | **1** | **2** | **3** | **4** | **5** | **6** | **7** | **8** | **9** | **10** | **11** |  |
| 1. Bendicksen D, 2022 | U | Y | Y | N | N | Y | Y | U | U | U | Y | Moderate |
| 1. De la Roche M, 2021 | U | Y | Y | N | N | Y | Y | U | U | U | Y | Moderate |
| 1. Morgan P, 2008 | U | Y | Y | Y | Y | Y | Y | U | U | U | Y | High |
| 1. Nabagiez, 2013 | U | Y | Y | N | N | Y | Y | U | U | U | Y | Moderate |
| 1. Nabagiez, 2016 | U | Y | Y | N | N | Y | Y | U | U | U | Y | Moderate |
| 1. Ranzenbach, 2012 | U | N | U | N | N | Y | Y | N | N | N | Y | Low |
| 1. Reed D, 2017 | U | Y | Y | Y | N | Y | Y | U | U | U | Y | Moderate |
| 1. Resnick C, 2016 | Y | Y | Y | N | N | Y | Y | U | U | U | Y | Moderate |
| 1. Singh, 2011 | Y | Y | Y | Y | Y | Y | Y | U | U | U | Y | High |
| 1. Timmermans M, 2017 | Y | Y | Y | Y | Y | Y | Y | U | U | U | Y | High |
| 1. Theunissen B, 2014 | U | Y | Y | N | N | Y | Y | U | U | U | Y | Moderate |
| 1. Moore J, 2021 | U | Y | Y | N | N | Y | Y | N | N | N | Y | Low |
| 1. Hains T, 2021 | U | Y | Y | Y | Y | Y | Y | U | U | U | Y | High |
| 1. Pavlik D, 2017 | U | Y | U | N | N | U | Y | U | U | U | Y | Low |
| 1. Mains C, 2009 | U | Y | Y | N | N | Y | Y | U | U | U | Y | Moderate |
| 1. Fung D, 2020 | Y | Y | Y | N | N | Y | Y | U | U | U | Y | Moderate |
| 1. Malloy S, 2021 | Y | Y | Y | N | N | Y | Y | U | U | U | Y | Moderate |
| 1. Senft J, 2019 | U | Y | Y | Y | Y | Y | Y | U | U | U | Y | High |
| 1. Rodi S, 2006 | Y | Y | Y | N | N | Y | Y | U | U | U | Y | Moderate |
| 1. Gibson K, 2023 | U | Y | Y | N | N | Y | Y | U | U | U | Y | Moderate |
| 1. Hascall R, 2018 | U | Y | Y | N | N | Y | Y | N | N | N | Y | Low |
| 1. Kumar R, 2013 | N | N | U | N | N | Y | Y | N | N | N | U | Low |
| 1. Ducharme J, 2009 | Y | U | Y | N | N | Y | Y | N | N | N | Y | Moderate |
| 1. Dies N, 2016 | U | U | Y | N | N | Y | Y | N | N | N | Y | Moderate |
| 1. Drennan V, 2015 | Y | U | Y | Y | Y | Y | Y | U | U | U | Y | Moderate |
| 1. Misurka J, 2023 | U | U | Y | N | N | Y | U | N | N | N | Y | Low |
| 1. Chao, 2017 | Y | Y | Y | N | N | Y | Y | N | N | N | Y | Moderate |
| 1. Odogwu S, 2024 | U | Y | Y | N | N | U | U | N | N | N | Y | Low |
| 1. Ononye R, 2024 | U | Y | Y | N | N | U | U | N | N | N | Y | Low |

Keys: Y: Yes; N: No; U: Unclear; n/a: not applicable

1. Were the two groups similar and recruited from the same population?
2. Were the exposures measured similarly to assign people to both exposed and unexposed groups?
3. Was the exposure measured in a valid and reliable way?
4. Were confounding factors identified?
5. Were strategies to deal with confounding factors stated?
6. Were the groups/participants free of the outcome at the start of the study (or at the moment of exposure)?
7. Were the outcomes measured in a valid and reliable way?
8. Was the follow up time reported and sufficient to be long enough for outcomes to occur?
9. Was follow up complete, and if not, were the reasons to loss to follow up described and explored?
10. Were strategies to address incomplete follow up utilized?
11. Was appropriate statistical analysis used?

##

## Supplementary file 4. Quality appraisal of a case series study

**JBI critical appraisal checklist for case series**

| **Study** | **JBI Appraisal items** | | | | | | | | | | **Score** |
| --- | --- | --- | --- | --- | --- | --- | --- | --- | --- | --- | --- |
|  | **1** | **2** | **3** | **4** | **5** | **6** | **7** | **8** | **9** | **10** |  |
| Althausen P, 2016 | U | Y | Y | Y | Y | N | U | Y | U | Y | Moderate |

1. Were there clear criteria for inclusion in the case series?
2. Was the condition measured in a standard, reliable way for all participants included in the case series?
3. Were valid methods used for identification of the condition for all participants included in the case series?
4. Did the case series have consecutive inclusion of participants?
5. Did the case series have complete inclusion of participants?
6. Was there clear reporting of the demographics of the participants in the study?
7. Was there clear reporting of clinical information of the participants?
8. Were the outcomes or follow up results of cases clearly reported?

##

## Supplementary file 5. Quality assessment of a case-control study

**JBI critical appraisal checklist for case-control studies**

| **Study** | **JBI Appraisal items** | | | | | | | | | | **Score** |
| --- | --- | --- | --- | --- | --- | --- | --- | --- | --- | --- | --- |
|  | **1** | **2** | **3** | **4** | **5** | **6** | **7** | **8** | **9** | **10** |  |
| Decloe M, 2015 | U | Y | Y | Y | Y | U | N | Y | U | Y | Moderate |

1. Were the groups comparable other than the presence of disease in cases or the absence of disease in controls?

2. Were cases and controls matched appropriately?

3. Were the same criteria used for identification of cases and controls?

4. Was exposure measured in a standard, valid and reliable way?

5. Was exposure measured in the same way for cases and controls?

6. Were confounding factors identified?

7. Were strategies to deal with confounding factors stated?

8. Were outcomes assessed in a standard, valid and reliable way for cases and controls?

9. Was the exposure period of interest long enough to be meaningful?

10. Was appropriate statistical analysis used?

## Supplementary file 6. Quality assessment of cross-sectional studies

**JBI critical appraisal checklist for cross-sectional studies**

| **Study** | **JBI Appraisal items** | | | | | | | | **Score** |
| --- | --- | --- | --- | --- | --- | --- | --- | --- | --- |
|  | **1** | **2** | **3** | **4** | **5** | **6** | **7** | **8** |  |
| Berg G, 2012 | Y | Y | Y | U | N | N | Y | Y | Moderate |
| Berkowitz O, 2020 | N | U | Y | U | N | N | Y | Y | Low |
| Burrows K, 2023 | Y | Y | Y | U | N | N | Y | Y | Moderate |
| Chatterjee S, 2018 | U | Y | Y | Y | N | N | Y | Y | Moderate |
| Doan Q, 2012 | Y | Y | Y | U | N | N | Y | Y | Moderate |
| Doan Q, 2013 | U | Y | U | Y | N | N | Y | Y | Low |
| Doan Q, 2013 | U | Y | U | Y | N | N | Y | Y | Low |
| Gifford A, 2011 | N | Y | U | U | N | N | Y | Y | Low |
| Griffith C, 2023 | N | U | U | Y | N | N | Y | Y | Low |
| Hains T, 2018 | Y | U | U | Y | N | N | U | Y | Low |
| Halvachizadeh S, 2022 | Y | Y | Y | Y | N | N | Y | Y | Moderate |
| Kuilman L, 2012 | Y | Y | Y | U | N | N | Y | Y | Moderate |
| Meijer K, 2017 | U | Y | Y | Y | N | N | Y | Y | Moderate |
| Korth M, 2022 | Y | Y | Y | Y | N | N | Y | Y | Moderate |
| Williams L, 2014 | U | Y | U | Y | N | N | Y | U | Low |
| Joyce P, 2018 | U | U | Y | Y | N | N | Y | Y | Moderate |
| Hooker R, 2010 | Y | U | Y | U | N | N | U | Y | Low |
| Bohm E, 2010 | U | U | Y | U | N | N | U | U | Low |

Key: Y – Yes; N – No; U – Unclear; n/a – not applicable

1. Were the criteria for inclusion in the sample clearly defined?
2. Were the study subjects and the setting described in detail?
3. Was the exposure measured in a valid and reliable way?
4. Were objective, standard criteria used for measurement of the condition?
5. Were confounding factors identified?
6. Were strategies to deal with confounding factors stated?
7. Were the outcomes measured in a valid and reliable way?
8. Was appropriate statistical analysis used?

## Supplementary file 7. Quality assessment of mixed-methods studies

**MMAT critical appraisal of mixed methods studies**

| **Study** | **MMAT**  **Mixed methods items** | | | | | **Score** |
| --- | --- | --- | --- | --- | --- | --- |
|  | **1** | **2** | **3** | **4** | **5** |  |
| Drennan V, 2018 | CT | Y | Y | CT | Y | Moderate |
| Halter M, 2020 | CT | Y | Y | CT | Y | Moderate |
| Hepp S, 2017 | N | Y | CT | CT | Y | Moderate |
| Kruk M, 2007 | N | CT | CT | N | CT | Low |
| McCord C, 2009 | Y | Y | Y | Y | Y | High |
| Tucker R, 2021 | CT | Y | Y | CT | Y | Moderate |
| White H, 2013 | CT | Y | CT | N | CT | Low |
| Kurti L, 2011 | CT | Y | CT | CT | Y | Moderate |
| Drennan V, 2020 | CT | Y | Y | CT | Y | Moderate |
| Farmer J, 2011 | CT | CT | N | N | CT | Low |

Y – Yes; N – No; CT – can’t tell.

1. Is there an adequate rationale for using a mixed methods design to address the research question?

2. Are the different components of the study effectively integrated to answer the research question

3. Are the outputs of the integration of qualitative and quantitative components adequately interpreted?

4. Are divergences and inconsistencies between quantitative and qualitative results adequately addressed

5. Do the different components of the study adhere to the quality criteria of each tradition of the methods involved?

## Supplementary file 8. Quality assessment of qualitative studies

**JBI critical appraisal checklist for qualitative studies**

| **Study** | **JBI Appraisal items** | | | | | | | | | | **Score** |
| --- | --- | --- | --- | --- | --- | --- | --- | --- | --- | --- | --- |
|  | **1** | **2** | **3** | **4** | **5** | **6** | **7** | **8** | **9** | **10** |  |
| Drennan V, 2011 | U | Y | Y | Y | Y | N | N | U | N | Y | Moderate |
| Halter M, 2017 | U | Y | Y | Y | Y | N | N | U | Y | Y | Moderate |
| Quick, 2013 | U | U | U | Y | Y | U | U | U | Y | Y | Low |
| Van Vught A, 2014 | U | Y | U | Y | U | N | N | N | N | U | Low |
| Taylor F, 2019 | Y | Y | Y | Y | Y | Y | Y | Y | Y | Y | High |
| Smalley S, 2020 | U | Y | Y | Y | Y | U | Y | U | Y | U | Moderate |
| Sellers C, 2022 | U | U | Y | Y | U | N | N | U | N | U | Low |
| Drennan V, 2017 | U | Y | Y | Y | Y | N | U | Y | Y | Y | Moderate |
| Bowen S, 2016 | U | Y | Y | Y | Y | N | N | U | Y | Y | Moderate |

1. Is there congruity between the stated philosophical perspective and the research methodology?

2. Is there congruity between the research methodology and the research question or objectives?

3. Is there congruity between the research methodology and the methods used to collect data?

4. Is there congruity between the research methodology and the representation and analysis of data?

5. Is there congruity between the research methodology and the interpretation of results?

6. Is there a statement locating the researcher culturally or theoretically?

7. Is the influence of the researcher on the research, and vice- versa, addressed?

8. Are participants, and their voices, adequately represented?

9. Is the research ethical according to current criteria or, for recent studies, and is there evidence of ethical approval by an appropriate body?

10. Do the conclusions drawn in the research report flow from the analysis, or interpretation, of the data?
